# Lophine analogues as fluorophores for selective bioimaging of the endoplasmic reticulum†

**DOI:** 10.1039/d4cc06552b

**Published:** 2025-03-17

**Authors:** Danica Drpic, Fabián A. Amaya-García, Miriam M. Unterlass

**Affiliations:** a CeMM Research Center for Molecular Medicine of the Austrian Academy of Sciences Lazarettgasse 14, AKH BT25.3 1090 Vienna Austria; b Department of Chemistry, Universität Konstanz Universitätsstrasse 10 78464 Konstanz Germany fabian.amaya-garcia@uni-konstanz.de; c Chair of Chemical Technology of Materials Synthesis, Julius Maximilian University Würzburg Röntgenring 11 97070 Würzburg Germany miriam.unterlass@uni-wuerzburg.de; d Fraunhofer Institute for Silicate Research Neunerplatz 2 97082 Würzburg Germany miriam.unterlass@isc.fraunhofer.de

## Abstract

The design of small-molecule fluorescent probes for labelling the endoplasmic reticulum (ER) revolves around a well-established albeit limited group of structural architectures. Here, we synthesized new fluorescent lophines in one step in high-temperature water (HTW) and explored their application as dyes for selective bioimaging of the ER.

## Abstract 2

We report a new set of synthetically accessible lophine analogues for selective live-cell imaging of the endoplasmic reticulum (ER). The ER selectivity is achieved without employing state-of-the-art moieties used for the design of ER-targeting dyes.

## Graphical abstract

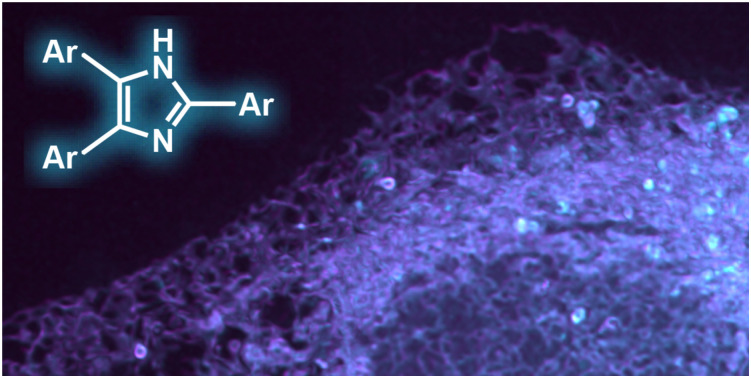

Imidazole and imidazole-containing compounds have been explored since the 19th century. The first synthesis of imidazole from glyoxal and ammonia was reported by Debus in 1858.^1^ Several name reactions towards imidazoles featuring different substitution patterns have been developed.^2^ In particular, Japp^3^ and Radziszewski^4^ independently reported the synthesis of different 2,4,5-triphenylimidazoles. These contributions^1,3,4^ are the pioneer reports of the Debus–Radziszewski multicomponent reaction (MCR), which is a widely known method to synthesize lophine (Fig. 1(A)) and analogues thereof.

**Fig. 1:**
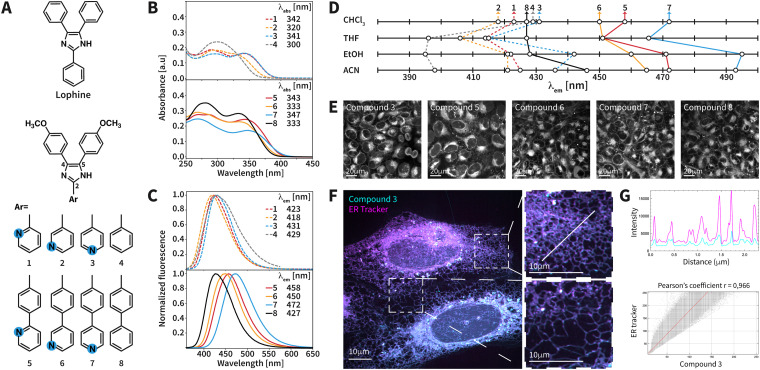
(A) Structures of lophine and its analogues 1–8. Absorption (B) and fluorescence emission (C) spectra of 1–8 in CHCl_3_ at 10 μM. The *λ*_abs_ for the longest absorption band and the *λ*_em_ are indicated in the top right corner of the spectra. (D) Recorded *λ*_em_ for 1–8 in different solvents (see the ESI† for full spectra). (E) Confocal microscopy images of U2OS cells treated with compounds 3 and 5–8 at 10 μM using *λ*_exc_ = 375 nm and *λ*_em_ = 465–530 nm. Scale bars: 20 μm. (F) High-resolution confocal microscopy images of U2OS cells treated with compound 3 (cyan) and the commercial ER-Tracker (magenta). Scale bars: 10 μm. (G) The upper graph shows colocalization signals between compound 3 (cyan) and the ER-Tracker (magenta). The lower graph presents quantification of colocalization, performed using the ImageJ JaCOP plugin, comparing the overlap signals between compound 3 and the ER-Tracker.

Lophine analogues have been investigated for their photophysical properties. In particular, several lophine analogues have been used as fluorescent probes, since their fluorescence properties change in response to the presence of chemical species or to fluctuations of the chemical environment.^5,6^ In that regard, applications dealing with bioimaging have been explored and the reported probes typically localize in the cytoplasm of the cell (see Table S1, ESI†). In contrast, selective labelling of cellular organelles has been rarely reported for lophine-based probes, with selective labelling of mitochondria as the reported main target organelle.^7,8^

Among the cellular organelles of eukaryotes, the endoplasmic reticulum (ER) is the largest and most dynamic membrane structure, extending throughout the cytoplasm. The ER originates from the nuclear envelope as stacks of sheets and transitions into a network of interconnected tubules and sheets at the cell's periphery.^9–11^ The ER plays a critical role in protein folding, post-translational modifications, ion storage, and the synthesis of lipids and steroids. More importantly, disruption in the ER function can contribute to the development of metabolic and cardiovascular diseases as well as cancer.^12,13^ Developing ER-selective fluorophores is therefore highly attractive to monitor the real-time dynamics and changes of this organelle, *e.g.*, morphology, *via* fluorescence microscopy.

State-of-the-art fluorescent probes for the ER are limited to a handful of established molecules and functional groups. The probes generally leverage fluorophores such as BODIPY, naphthalimide, and coumarin, whereas ER selectivity is achieved through functionalization with moieties such as sulfonamide, sulfonylurea, or perfluorophenyl.^14,15^ In general, sulfonamide and sulfonylurea groups are expected to endow probes with affinity for receptors of ATP-sensitive K^+^ channels in the ER. Nonetheless, this action mechanism might also perturb the normal ER function. Alternatively, naphthalimides^16^ and styryl-containing compounds,^17^ which do not feature sulfonamide groups, have been reported for ER labelling. The selectivity of such compounds has been ascribed to their lipophilicity. Nonetheless, multistep synthesis and laborious purification procedures are required to obtain these probes, which inherently challenges straightforward applicability in comparison to commercially available alternatives. In summary, ER selective fluorophores featuring structural novelty, synthetic accessibility, and biocompatibility are highly desired albeit scarce. Here, we complement the available toolbox of fluorophores for selective ER labelling with a set of new lophine analogues. The compounds (i) are synthesized in only one step *via* a recently described Debus–Radziszewski MCR in HTW as a medium,^18^ (ii) are obtained from commercially available starting materials, (iii) feature low toxicity and good cell permeability, and (iv) do not label further cellular organelles such as lysosomes or mitochondria. Interestingly, the compounds feature neither state-of-the-art fluorophores nor functional groups conventionally used for inducing ER-selectivity in dyes.

The synthesized compounds 1–8 are depicted in Fig. 1(A). All the compounds feature an architecture based on an imidazole ring substituted with 4-methoxyphenyl groups at positions 4 and 5. The compounds differ in the substituent at position 2. Compounds 1–3 bear a pyridine ring at position 2, which is connected to the imidazole through a single bond. Note that the connectivity with respect to the pyridine's nitrogen atom is different, and hence 1–3 are positional isomers. Compounds 5–7 feature a benzene ring as a spacer between the imidazole and the pyridine ring. Thus, these compounds are analogues of 1–3 that exhibit extended π-conjugation. Analogously to compounds 1–3, 5–7 are also positional isomers with respect to the pyridine ring. Compounds 4 and 8 feature phenyl and biphenyl substituents, respectively, at position 2. These compounds are benchmarks with respect to the compounds bearing a pyridine ring at position 2, *i.e.*, 4 as a benchmark for 1–3 and 8 as a benchmark for 5–7. Overall, the considered structural features give insights into the physicochemical properties in regard to (i) extended π-conjugation, (ii) the presence of a pyridine ring, and (iii) positional isomerism of the pyridine ring.

Compounds 1 to 8 were synthesized *via* the Debus–Radziszewski MCR between 4,4′-dimethoxybenzil and aromatic aldehydes using urea in HTW (see the ESI† for experimental details). State-of-the-art Debus–Radziszewski MCRs are typically performed in volatile organic compounds (VOCs) as solvents, use ammonium acetate as the N-source, and heavily rely on catalysts.^2^ The synthesis employed in this work targets sustainability in several ways. First, the use of VOCs as solvents is prevented. HTW exhibits decreased polarity compared to r.t. water. Therefore, selecting the appropriate temperature (*T*) allows for matching the polarity of VOCs used as solvents and dissolve low-polarity starting materials in nothing but HTW. Second, urea is used as a non-hygroscopic, non-toxic, and available N-source to propel the imidazole formation. Third, the synthesis is free from additional catalysts, enabled through HTW itself providing increased Brønsted acido-basicity. Therefore, waste generated from the synthesis of reaction catalysts is completely avoided. Such an interplay between polarity and Brønsted acido-basicity has allowed for synthesizing several classes of compounds, such as imides,^19^ perinones,^20^ benzimidazoles,^21,22^ and quinoxalines.^23,24^ Fourth, straightforward purification procedures can be implemented. All compounds were isolated *via* filtration and purified through washing steps or recrystallization. Compounds 1–4 and 8 were previously obtained,^18^ while compounds 5–7 are reported here for the first time. The compounds were obtained with yields ranging from 62 to 85% and the structures were confirmed through ^1^H and ^13^C NMR spectroscopy and electrospray ionization high-resolution mass spectrometry (ESI-HRMS).

After synthesizing the compounds, we evaluated their photophysical properties. Absorption spectra were measured in CHCl_3_, tetrahydrofuran (THF), ethanol (EtOH), and acetonitrile (ACN). Irrespective of the solvent, all compounds exhibit at least one absorption band in the range of 250–360 nm (Fig. S2, ESI†). We hypothesize that these bands correspond to π–π* transitions arising from the triphenylimidazole moiety.^25^ For all the compounds containing a pyridine ring and 8, one absorption band can be observed in the region of 300–360 nm. The position of this band is more sensitive to the structural features. In CHCl_3_ (Fig. 1(B)), compound 8 (*λ*_abs_ = 333 nm) exhibits *λ*_abs_ longer than that of compound 4 (*λ*_abs_ = 300 nm). This is consistent with the extended π-conjugation of 8. Nevertheless, compounds 1–3 exhibit *λ*_abs_ comparable to that of their analogues featuring extended π-conjugation, *e.g.*, *λ*_abs_(1) = 342 nm and *λ*_abs_(5) = 343 nm (Fig. 1(B)). This is mainly observed in CHCl_3_. The *λ*_abs_ of compounds 1–4 was shorter than that of their analogues featuring extended π-conjugation in all the other tested solvents (Table S2, ESI†). Furthermore, all the pyridine-containing compounds show *λ*_abs_ comparable to or longer than that of their benchmark compound in CHCl_3_ (Fig. 1(B)). With respect to the pyridine's substitution pattern, compounds 1 and 3 featuring 2-substituted and 4-substituted pyridine, respectively, show *λ*_abs_ longer than that of compound 2, which contains a 3-substituted pyridine (Fig. 1(B)). This was also observed among the isomeric compounds 5–7. For spectra measured in THF, EtOH and ACN, the longest *λ*_abs_ exhibited changes similar to those in CHCl_3_ with respect to the presence of the pyridine ring and its substitution.

Next, we recorded fluorescence emission spectra. The selected solvents feature different dielectric constants (*ε*(CHCl_3_) = 4.8, *ε*(THF) = 7.6, *ε*(EtOH) = 24.5, *ε*(ACN) = 37.5), and hence give insight into the fluorescence properties under environments of different polarity. The longest *λ*_abs_ of each compound was used as the excitation wavelength. In CHCl_3_, the emission maximum (*λ*_em_) of the compounds ranges from 418 nm (compound 2) to 472 nm (compound 7) (Fig. 1(C) and (D)). This range broadens upon increasing the polarity of the solvent. In ACN (Fig. 1(D)), the *λ*_em_ ranges from 398 nm (compound 4) to 493 nm (compound 7). In CHCl_3_, compounds 5–8 exhibit *λ*_em_ values similar to or longer than those of their corresponding analogues 1–4 (Fig. 1(C)). This was also observed in THF and ACN. As an exception, we observed that compound 3 features a longer *λ*_em_ (442 nm) than compound 8 (428 nm) in EtOH despite the extended π-conjugation of 8 (Fig. 1(D)). Different trends for *λ*_em_ are observed with respect to the pyridine ring. Compounds 1–3 and benchmark compound 4 do not show considerably different *λ*_em_ in CHCl_3_, while increasing the polarity of the solvent allows for observing different *λ*_em_ (Fig. 1(D)). Furthermore, compounds 5–7 feature *λ*_em_ values longer than that of their benchmark compound 8 in all the employed solvents. With respect to the substitution of the pyridine ring, we observed changes in *λ*_em_. Compounds 1–3 exhibit comparable *λ*_em_ in CHCl_3_, while differences are observed in ACN (Fig. 1(D)). In contrast, compounds 5–7 generally exhibit different *λ*_em_ in all the tested solvents. Among compounds with a comparable length of π-conjugation, the isomer containing a 4-substituted pyridine ring (3 and 7) tends to exhibit a long *λ*_em_, followed by 2-substituted (1 and 5) and 3-substituted pyridine (2 and 6). This can be clearly seen in solvents of increased polarity (EtOH and ACN; Fig. 1(D)). Overall, compound 7 consistently exhibits the longest *λ*_em_ in all the tested organic solvents. The quantum yields (*ϕ*_F_) in THF ranged from 0.39 to 0.62 (Table S3, ESI†). Under aqueous conditions, 1–8 are soluble at 10 μM (Fig. S3, ESI†) and the *ϕ*_F_ drops to 0.31 or lower. The influence of the pH on the photophysical properties was investigated (Fig. S4, ESI†). The *λ*_abs_ shifts upon decreasing the pH. The differences between the *λ*_abs_ at pH = 6.9 and 3.6 are not longer than 22 nm for most of the compounds. Compound 3 is an exception, featuring *λ*_abs_ = 334 nm at pH = 6.9 and *λ*_abs_ = 408 nm at pH = 3.6. Furthermore, decreasing the pH to 4.3 resulted in shifts of *λ*_em_ between 6 and 20 nm and enhanced fluorescence for most of the compounds. Only compound 4 exhibits *λ*_em_ = 416 nm (pH = 6.9) and a new *λ*_em_ = 457 nm (pH = 3.6). The shifts result from the protonation of basic nitrogen atoms in the pyridine and imidazole rings. Overall, values of pH ≤ 5.2 are required to shift the *λ*_abs_ and *λ*_em_.

We next tested compounds 1–8 as dyes for bioimaging, including lophine (Fig. 1(A)), for comparison purposes. We evaluated the cytotoxicity of the compounds using the CellTiter-Glo luminescence cell viability assay. This measures ATP levels as an indicator of metabolically active cells. Human osteosarcoma (U2OS) cells were treated with the compounds at 10 μM for 50 hours. The cell survival rates ranged from 69% (compound 1) to 100% (compound 8), suggesting low cytotoxicity (Table 1). U2OS cells displayed high fluorescence intensity within 5 minutes of treatment (Fig. 1(E) and Fig. S6, ESI†). Compounds 1, 2, and 4 and lophine displayed weak fluorescence, which is suspected to arise from autofluorescence. In contrast, compounds 3 and 5–8 exhibited high fluorescence (Table 1 and Fig. S5, S6, ESI†). U2OS cells incubated in a medium with 10% fetal bovine serum and treated with the compounds resulted in a 4- to 8-fold fluorescence intensity without changes in spectral shape (Fig. S7 and S8, ESI†). Compounds 3 and 5–8 were selected to conduct further bioimaging experiments because of their high fluorescence. Specific labelling of cellular organelles in U2OS cells was examined through colocalization experiments. In comparison to ER-Tracker Red (ER-Tracker), the Pearson correlation coefficient (PCC) values of the compounds ranged from 0.91 to 0.98 (Table 1 and Fig. S9, S13A, ESI†). Moreover, the dyes showed low PCCs with respect to MitoTracker Deep Red 633 (PCC = 0.65–0.67) and LysoTracker Red DND-99 (PCC = 0.30–0.47) (Fig. S10, S11, and S13B, C, ESI†). Colocalization against Golgi tracker BODIPY TR ceramide resulted in high PCC values (PCC = 0.82–0.94) (Fig. S12 and S13D, ESI†). Yet, Golgi tracker also exhibited non-specific staining of the ER and cell membrane (Fig. S14, ESI†) and we suspect that this influences the PCC for Golgi. While we cannot completely rule out that our compounds do not label the Golgi apparatus, the ER selectivity is clear.

**Table 1:** Evaluation of cytotoxicity and selected parameters from fluorescence microscopy experiments for compounds 1–8 and lophine

Compound	Cell viability^a^ (%)	*λ*_em_ b	ER PCC^c^
1	69	—	—
2	83	—	—
3	70	450	0.98
4	71	—	—
5	79	460	0.98
6	90	460	0.95
7	70	480	0.97
8	100	420	0.91
Lophine	99	—	—

a Expressed as % with respect to DMSO as a control.

b Emission maximum observed *via* optical microscopy with *λ*_exc_ = 375 nm.

c The Pearson correlation coefficient for the endoplasmic reticulum.

Lophine did not prove to be well-suited for bioimaging under the employed conditions. In contrast, compounds 3 and 5–8 exhibit a high PPC for the ER. Since 3 features π-conjugation shorter than 5–8, we hypothesize that extended π-conjugation is not fully essential for ER staining. Note that our compounds mainly feature extended π-conjugation at position 2 of the imidazole. Benchmark compound 8 exhibits a high PCC for the ER, which suggests that the pyridine ring in 5–7 is not essential for the selectivity. Among compounds 1–4, we could only establish selective ER labeling for compound 3. Yet, compounds 1, 2, and 4 are weakly fluorescent, and we cannot conclude the lack of selectivity based on fluorescence microscopy. We hypothesize that, instead of triggering selectivity, the 4-substituted pyridine endows compound 3 with fluorescence properties more suitable than those of 1, 2, and 4.

Commercial ER dyes exhibit changes in subcellular localization depending on their concentration. For instance, DiOC6(3) shows specificity for mitochondria at lower concentrations and for the ER at higher concentrations.^26^ In contrast, our compounds exhibit consistent ER staining at both micromolar and nanomolar concentrations (Fig. S15, ESI†). This concentration-independent staining is expected to be useful for retaining ER-specificity when a low concentration of the dyes is desired.

We conducted further bioimaging experiments with compound 3 because of the high PCC for the ER and intense fluorescence. The compound was photostable when imaged for 1 h every 1 min (Fig. S17, ESI†). Moreover, compound 3 was used for higher-resolution imaging in a confocal microscope equipped with a SoRa Super Resolution Spinning Disk (150 nm resolution). This method optically improves lateral resolution by a factor of 1.37 with a single exposure.^27^ High-resolution images showed an interconnected network of tubules and membranes, characteristic of the ER (Fig. 1(F), (G) and Fig. S16, ESI†).

In summary, we described the synthesis of new fluorescent lophine analogues applicable for live-cell imaging of the ER. Overall, selective labeling of the ER arises, but not exclusively, in compounds featuring extended π-conjugation and does not depend on the presence of the pyridine ring. We suspect that the lipophilic character of the compounds is the driving force for the selectivity. We currently focus on understanding the mechanism behind the selective ER labelling as well as developing analogues with long excitation wavelengths and tunable emission.

D. Drpic: cytotoxicity assays, fluorescence microscopy. F. A. Amaya-Garcia: synthesis, structural characterization, study of photophysical properties. M. M. Unterlass: supervision, funding. The manuscript was written and reviewed by all authors.

This work was funded by the Vienna Science and Technology Fund (WWTF; grant # LS17-051) and the Austrian Science Fund (FWF; grant # START Y1037-N28). We thank the Core Imaging facility of the Medical University of Vienna for access to fluorescence microscopes.

## Data availability

The data supporting this article have been included as part of the paper and in the ESI† of the article.

## Conflicts of interest

There are no conflicts to declare.

## Supplementary Material

CC-061-D4CC06552B-s001
