# Supplementary material for: Lophine analogues as fluorophores for selective bioimaging of the endoplasmic reticulum
Source: Chem Commun (Camb). 2025 Mar 17;61(28):5293–6. doi: 10.1039/d4cc06552b (PMC11911997; doi:10.1039/d4cc06552b)
Supplement: CC-061-D4CC06552B-s001 [file CC-061-D4CC06552B-s001.pdf]

Supplementary Information (SI) for ChemComm.
This journal is © The Royal Society of Chemistry 2025

Electronic Supporting Information (ESI)

Lophine analogues as fluorophores for selective bioimaging of the endoplasmic reticulum.

Danica Drpic,[a,†] Fabián A. Amaya-García,*[a,b,†] Miriam M. Unterlass.*[a,b,c,d]

[a]CeMM Research Center for Molecular Medicine of the Austrian Academy of Sciences, Lazarettgasse 14, AKH BT25.3, 1090 Vienna, Austria.
[b]Universität Konstanz, Department of Chemistry, Universitätsstrasse 10, 78464 Konstanz, Germany.
[c] Chair of Chemical Technology of Materials Synthesis, Julius Maximilian University, Würzburg, Röntgenring 11, 97070 Würzburg, Germany.
[d]Fraunhofer Institute of Silicate Research, Neunerplatz 2, 97082 Würzburg, Germany.
[†]The authors contribute equally to this work.
*miriam.unterlass@uni-wuerzburg.de, miriam.unterlass@isc.fraunhofer.de, fabian.amaya-garcia@uni-konstanz.de

1. General methods

Chemicals and Synthesis: All the chemicals were obtained from TCI Germany or Sigma Aldrich and used without further purification. The reactions were conducted in a microwave reactor Anton Paar Monowave 450, using G30 and G10 glass vials. Distillated water was employed as solvent unless otherwise is stated. ^{1}H and ^{13}C NMR spectra were recorded on a Bruker Avance III 400 spectrometer. Samples were dissolved in DMSO-d_6 (δ_H = 2.50). The solvent residual signal as reference for reporting the chemical shifts. The coupling constants are reported in Hertz. UV-Vis spectra were recorded in a DS5 double beam UV-Vis spectrophotometer (Edinburgh Instruments) and fluorescence emission spectra were recorded in a Cary Eclipse fluorescence Spectrophotometer (Agilent Technologies). Samples were prepared in solvents of spectroscopic quality.

Cell Culture and Imaging: U2OS cells were cultured in DMEM 1x GlutaMax-I (Gibco, Cat no. 31966-021) medium supplemented with 10% FBS (Gibco, Cat no. 10270-106) and 1% Penicillin-Streptomycin (Gibco, 15140-122). The cells were maintained under standard conditions at 37°C in a humidified atmosphere containing 5% CO_2.

U2OS cells were seeded in Ibidi dishes (u-slide 8 well glass bottom, Cat. no. 80827) at a density of 100,000 cells per well. The following day, the cells were stained with commercial ER- tracker (Invitrogen, ER-tracker, BODIPY-TR (glibenclamide), Cat. no. E342550, Lot 2559188), MitoTracker Deep Red 633 (Invitrogen, Cat no. M22426, Lot 3986A) at a dilution of 1:1000 or 100nM Lysotracker Red DND- 99 (Invitrogen, Ref 7528, Lot 1971192) BODIPY TR- ceramide (Invitrogen, ref 7540, Lot 2790115) at concentration 2µM or 5µM.

Compounds **1**-**8** and lophine were added at a concentration of 10 µM, 30 minutes before imaging.

Images were acquired using the Olympus IXplore SpinSR, equipped with a 60x silicone oil objective (NA 1.3) and SoRa Super Resolution Spinning Disk, providing a resolution of 150 nm. The acquisition was performed using cellSens Dimensions Software, and the images were processed through constrained iterative deconvolution for restoration.

A Z stack with a step size of 0.5 µm was used. The commercial ER tracker was excited at 561 nm, and compounds **1**-**8** were excited at 405 nm or 488nm.

Opera High content Imaging: U2OS cells were seeded in PhenoPlate™ 96-well microplates (Revvity, Cat. no. 6055302) at a concentration of 10,000 cells per well. The following day, the cells were treated with 10 µM of the corresponding compounds and incubated for 20 minutes before imaging.

Imaging was performed using the Opera Phenix™ High-Content Screening System with a 40x water immersion objective. Images were acquired using excitation light at 375 nm with emission at 435-480 nm, and excitation at 425 nm with emission at 465-530 nm. The acquired images were subsequently analyzed using ImageJ software, which included steps for background subtraction, intensity measurement, and quantification of fluorescence signals.

Image analysis: The correlation between two image channels (signals from compound **1**-**8** and ER-tracker, Mytotracker, Lysotracker, and Golgi tracker) was measured using Pearson's correlation coefficient (PCC). Colocalization analysis was performed using the JaCOP plugin in ImageJ, where a PCC of +1 indicates perfect positive correlation (high colocalization), 0 indicates no correlation (random distribution), and -1 indicates perfect negative correlation (mutual

exclusion). For the photostability assay fluorescence intensity was measured in the whole field of view over time using ImageJ.

Cell cytotoxicity assay: U2OS cells were seeded into PhenoPlate™ 96-well microplates (Revvity, Cat. No. 6055302) at a density of 10,000 cells per well. The following day, the cells were treated with 10 µM of the respective compound and maintained under standard conditions at 37°C in a humidified atmosphere containing 5% CO_2 for 50 hours.
Cell viability was assessed using the CellTiter-Glo® 2.0 Cell Viability Assay (Promega, Cat. No. G924A) according to the manufacturer's instructions. The reagent was equilibrated to room temperature, and an equal volume of CellTiter-Glo® Reagent was added to each well. The mixture was gently agitated on an orbital shaker for 2 minutes to induce cell lysis. Afterward, the plate was incubated for 10 minutes at room temperature to allow the luminescence signal to stabilize prior to measurement. This protocol ensures accurate assessment of cell viability by measuring ATP levels, indicative of metabolically active cells, providing reliable data for evaluating the effects of the compound treatment on U2OS cells.
Luminescence intensity was recorded using a SpectraMax i3x Multi-Mode Microplate Reader (Molecular Devices) with SoftMax Pro 7.0 software, following the CellTiter-Glo® Luminescence protocol.

Biological stability of the dyes in serum: U2OS cells were seeded and incubated in cell media containing either 10% FBS or no serum for 20 hours before staining with 10mM of each corresponding compound. Fluorescence intensity spectra were measured using a spectrophotometer with excitation fixed at 375 nm, recording emission from 405 nm to 580 nm. Additionally, the stability of the compounds in BSA or FBS was measured without the presence of cells. Fluorescence spectra were measured in PBS containing serial dilutions of FBS (10%, 5%, 2.5%, 1.25%, and 0.625%) mixed with 10 µM of the corresponding compound or in PBS containing serial dilutions of BSA (Albumin Fraction V, Roth, Art. Nr. 8076.4) (10 mg/mL, 5 mg/mL, 2.5 mg/mL, 1.25 mg/mL, and 0.625 mg/mL).

2. Database search of lophines and fluorescent probes.

We conducted a search in the SciFinder database on September 2024, employing the following criteria: imidazole as substructure and bioimaging as Abstract/Keywords. Dias et. al recently published a remarkable review on aryl-phenanthroimidazoles used as fluorescent probes (https://doi.org/10.1021/acssensors.2c01687). Moreover, we manually verified all the references and Table S1 includes specific references dealing with lophines used for bioimaging applications. Therefore, we did not include references dealing with lophine analogues and fluorescent probes. We include a representative structure, comments addressing the tested cell lines, and organelle localization.

Table S1. Fluorescent probes based on lophines for bioimaging applications.

**Representative molecule**	**Comment**	**Reference**
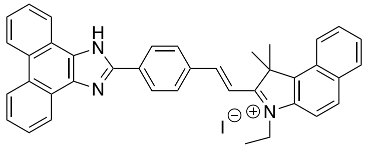	Bioimaging of BEL-7402 cells.<br>The compound localizes in the mitochondria.<br><br>Bioimaging of HeLa cells.<br>The compound is used for sensing of SO_3^-	https://doi.org/10.1016/j.talanta.2018.08.059<br><br><br>https://doi.org/10.1016/j.saa.2019.117788
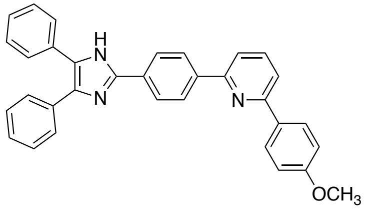	Bioimaging of *E. coli*. The authors comment that the binding mode of the fluorophore with the bacteria could be hydrogen bond interaction between imidazole with aminoacid groups of peptidoglycan cell wall of bacteria.	https://doi.org/10.1016/j.jphotobiol.2013.08.016
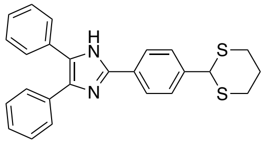	Bioimaging of *E. coli* and HeLa cells.<br>The compound is used for sensing of Hg^{2+}	https://doi.org/10.1039/D3SD00146F

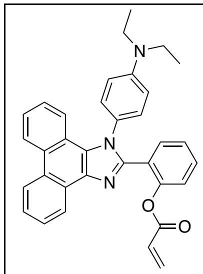	Bioimaging of HeLa cells.<br>The compound is used for sensing of cysteine	https://doi.org/10.1016/j.snb.2016.04.054
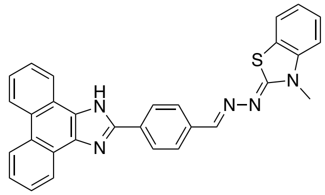	Bioimaging of MCF-7 cells and Zebrafish.<br>The compound is used for sensing of Hg^{2+} in a ratiometric fashion	https://doi.org/10.3390/molecules24122268
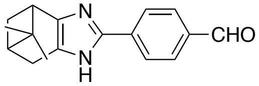	Bioimaging of HeLa cells and Zebrafish.<br>The compound is used for sensing of malonitrile	https://doi.org/10.1016/j.saa.2024.124476
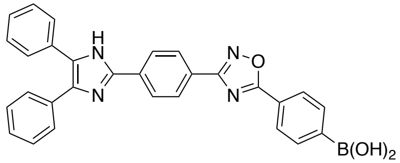	Bioimaging of HeLa cells<br>The compound is used for sensing of Fe^{3+} and F^-	https://doi.org/10.1016/j.aca.2019.03.040
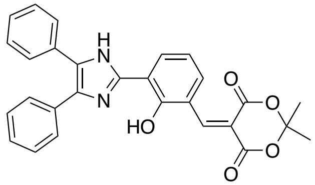	Bioimaging of *E. coli*.<br>The compound is used for sensing of CN^-	https://doi.org/10.1016/j.jphotochem.2022.114269
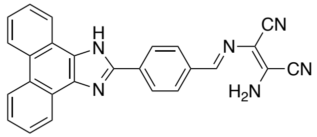	Bioimaging of HeLa cells.<br>The compound is used for sensing ClO^-	https://doi.org/10.1016/j.snb.2016.10.092

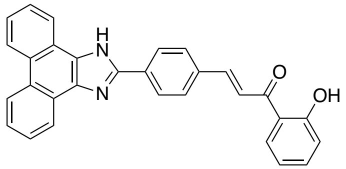	Bioimaging of HeLa cells.<br>The compound is used for sensing hydrazine	https://doi.org/10.1016/j.talanta.2017.12.039
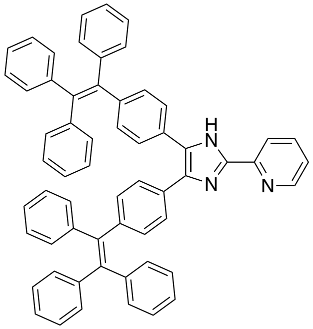	Bioimaging of B-16 cells	https://doi.org/10.1002/chem.202203772
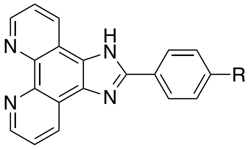	Bioimaging of HepG2 cells.<br>The compound localizes uniformly in the cytoplasm.	https://doi.org/10.1039/C2TC00175F
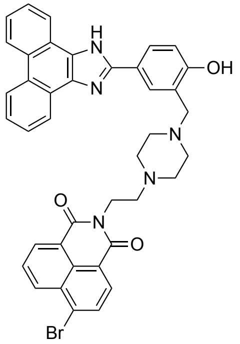	Bioimaging of HeLa cells<br>The compound is used for sensing Cu^{2+}	https://doi.org/10.1016/j.jinorgbio.2021.111466

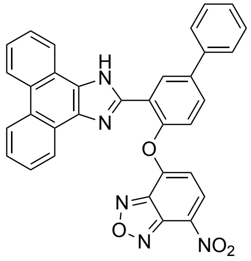	Bioimaging of HeLa cells and Zebrafish<br>The compound is used for sensing cysteine	https://doi.org/10.1039/C8TB02880J
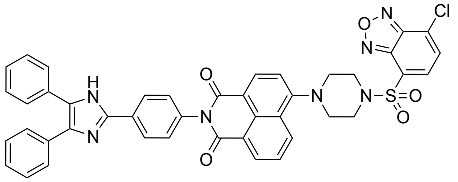	Bioimaging of HeLa cells<br>The compound is used for sensing cysteine	https://doi.org/10.1016/j.saa.2021.120409
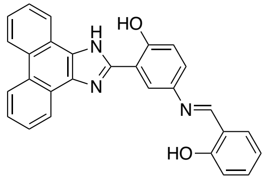	Bioimaging of HeLa cells<br>The compound is used for sensing Hg^{2+} and further sensing of S^{2-}	https://doi.org/10.1016/j.jphotochem.2019.112165
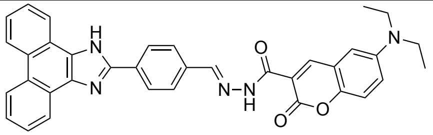	Bioimaging of HeLa cells and Zebrafish<br>The compound is used for sensing Cu^{2+}	https://link.springer.com/article/10.2116/analsci.20P366
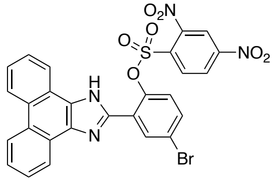	Bioimaging of HeLa cells<br>The compound is used for sensing thiophenol	https://doi.org/10.1016/j.jhazmat.2024.133464
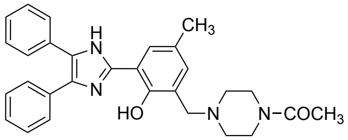	Bioimaging of A549 cells<br>The compound is used for sensing Sn^{2+}	https://doi.org/10.1016/j.molstruc.2023.136249

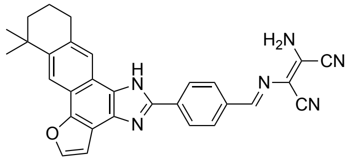	Bioimaging of MCF-7 cells and zebrafish<br>The compound is used for sensing ClO^-	https://doi.org/10.1016/j.jphotochem.2022.114533

3. Synthesis of compounds 1-8.

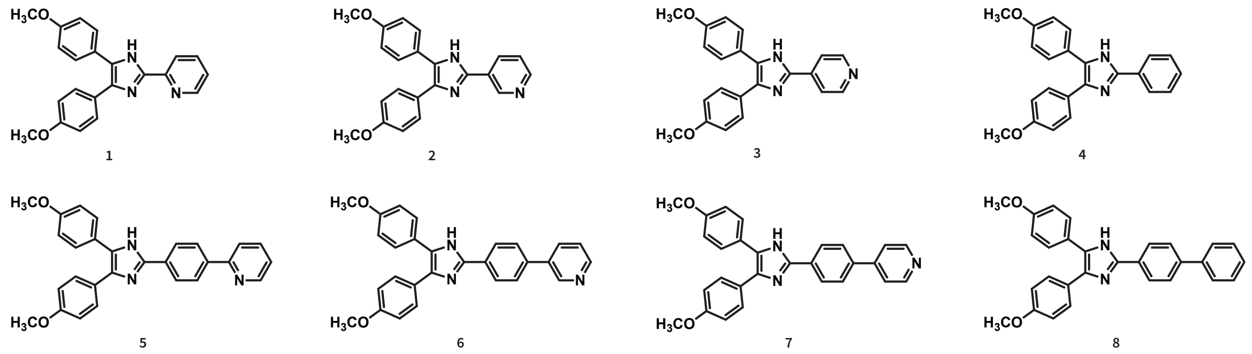

Figure S1. Structures of all the compounds in this work.

The synthesis and structural characterization of compounds **1**-**4** and **8** was previously reported by our group (*Green Chem.*, 2024, **26**, 10411-10421). The synthesis of compounds **5**-**7** was conducted following the reported conditions with slight modifications. The reactions were conducted in an Anton Paar Monowave 450 microwave reactor. In G30 microwave glass vial with a magnetic stirrer, 4,4'-dimetheoxybenzil (1.2 mmol), the corresponding aryl aldehyde (1.2 mmol), and urea (577 mg, 9.6 mmol) were suspended in 6.0 mL of distilled water. Then, the vial was place in the cavity of the reactor. The vial is first heated as fast as possible to 190 °C, at 1000 rpm and 400 W of power. The target temperature was reached and kept for 2 hours. After that time, the vial is cooled down to room temperature. Solid products can be distinguished from the aqueous phase after reaching room temperature. The solids were filtered and further purification was conducted for each compound as indicted below.

Compound **5**: Crude product was suspended in a mixture H_2O/EtOH 1/9 (4 mL) and heated up in a G10 microwave glass vial to 170 °C. This temperature was kept for 5 minutes and then the vial was cooled to room temperature. The obtained solution is allowed to settle at room temperature, treated with distilled water (3.0 mL), and allowed to settle at room temperature overnight. A yellow solid is formed. The solid is filtered, washed with mixture H_2O/EtOH 9/1 (3 mL), and dried in at 75°C to yield **5** as yellow solid (457.6 mg, 88% yield).

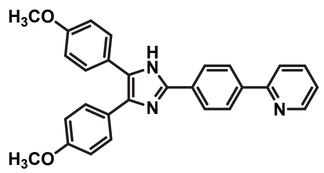

5

^{1}H NMR (400 MHz, DMSO-d_6) 12.62 (s, 1H), 8.69 (m, 1H), 8.19 (m, 4H), 8.03 (d, J = 8.0 Hz, 1H), 7.89 (td, J = 7.7, 1.9 Hz, 1H), 7.49 (d, J = 8.3 Hz, 2H), 7.44 (d, J = 8.3 Hz, 2H), 7.36 (dd, J = 7.5, 4.8 Hz, 1H), 7.02 (d, J = 8.3 Hz, 2H), 6.89 (d, J = 8.3 Hz, 2H), 3.80 (s, 3H), 3.76 (s, 3H);^{13}C NMR (100 MHz, DMSO-d_6) 158.8, 158.0, 155.5, 149.6, 144.4, 137.9, 137.2, 136.8, 131.0, 129.7, 128.2, 127.8, 127.6, 126.8, 125.3, 123.4, 122.6, 120.2, 114.1, 113.7, 55.2, 55.0; HRMS (ESI): m/z calcd for $C_{28}H_{24}N_3O_2$ $[M+H]^+$: 434.1863, found 434.1862.

Compound **6**: Crude product was suspended in a mixture H_2O/EtOH 1/9 (4 mL) and heated up in a G10 microwave glass vial to 170 °C. This temperature was kept for 5 minutes and then the vial was cooled to room temperature. The obtained solution is treated with 500 μL of distilled H_2O and allowed to settle at room temperature overnight. A pale yellow solid is formed after this step. The solid is filtered, washed with mixture H_2O/EtOH 1/1 (3 mL), and dried at room temperature to yield **6** as yellow solid (460.8mg, 89%).

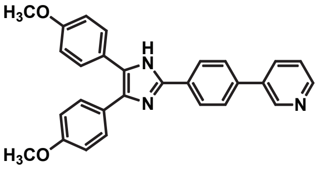

6

^1H NMR (400 MHz, DMSO-d_6) 12.61 (s, 1H), 8.98 (d, *J* = 2.3 Hz, 1H), 8.58 (dd, *J* = 4.7, 1.6 Hz, 1H), 8.19 (d, *J* = 8.3 Hz, 2H), 8.15 (d, *J* = 8.1 Hz, 1H), 7.86 (d, *J* = 8.4 Hz, 2H), 7.50 (m, 3H), 7.44 (d, *J* = 8.3 Hz, 2H), 7.02 (d, *J* = 8.5 Hz, 2H), 6.89 (d, *J* = 8.4 Hz, 2H), 3.80 (s, 3H), 3.75 (s, 3H); ^{13}C NMR (100 MHz, DMSO-d_6) ^{13}C NMR (100 MHz, DMSO-d_6) 158.8, 158.0, 148.6, 147.5, 144.3, 136.7, 136.3, 135.0, 133.9, 130.2, 129.8, 128.2, 127.8, 127.5, 127.1, 125.7, 123.9, 123.4, 114.1, 113.7, 55.2, 55.0; HRMS (ESI): m/z calcd for $C_{28}H_{24}N_3O_2$ $[M+H]^+$: 434.1863, found 434.1857.

Compound **7**: Crude product was suspended in a mixture H_2O/EtOH 1/9 (3 mL) and heated up in a G10 MW to 170 °C. This temperature was kept for 5 minutes and then the vial was cooled to room temperature. After this, an orange solid is formed. The solid is filtered and washed with H_2O/EtOH 1/1 (3 mL). The solid was dried in the oven at 75°C to yield **7** as orange solid (344.5 mg, 66% yield).

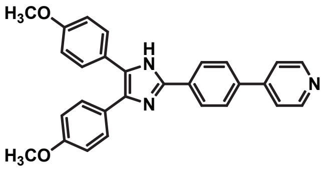

7

^1H NMR (400 MHz, DMSO-d_6) 12.65 (s, 1H), 8.65 (d, *J* = 6.2 Hz, 1H), 8.21 (d, *J* = 8.4 Hz, 2H), 7.93 (d, *J* = 8.3 Hz, 2H), 7.78 (d, *J* = 6.3 Hz, 2H), 7.49 (d, *J* = 8.3 Hz, 2H), 7.44 (d, *J* = 8.2 Hz, 2H), 7.02 (d, *J* = 8.3 Hz, 2H), 6.89 (d, *J* = 8.2 Hz, 2H), 3.80 (s, 3H), 3.75 (s, 3H); ^{13}C NMR (100 MHz, DMSO-d_6) ^{13}C NMR (100 MHz, DMSO-d_6) 158.9, 158.0, 150.3, 146.3, 144.1, 136.8, 136.2, 131.2, 129.8, 128.2, 127.73, 127.68, 127.1, 125.6, 123.4, 120.9, 114.1, 113.7, 55.2, 55.0; HRMS (ESI): m/z calcd for $C_{28}H_{24}N_3O_2$ $[M+H]^+$: 434.1863, found 434.1859.

The purity of compounds **1**-**8** was measured via quantitative ^1H NMR spectroscopy using dimethylsulfone as internal standard. The compounds and the standard were dissolved in deuterated DMSO (0.8-1.0 mL). Clear solutions were obtained and transferred to the NMR tube for measuring under the following conditions: 32 scans, acquisition time: 3.2768 s, relaxation delay (D_1): 30 s. Then, the purity of the compounds was calculated as indicated by Pauli *et al.* (*J. Med. Chem.* 2014, 57, 22, 9220–9231). The purities of the compounds were: **1**: 91%, **2**: 98%, **3**: 90%, **4**: 92%, **5**: 99%, **6**: 99%, **7**: 99%, **8**: 97%.

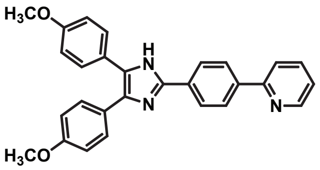

5

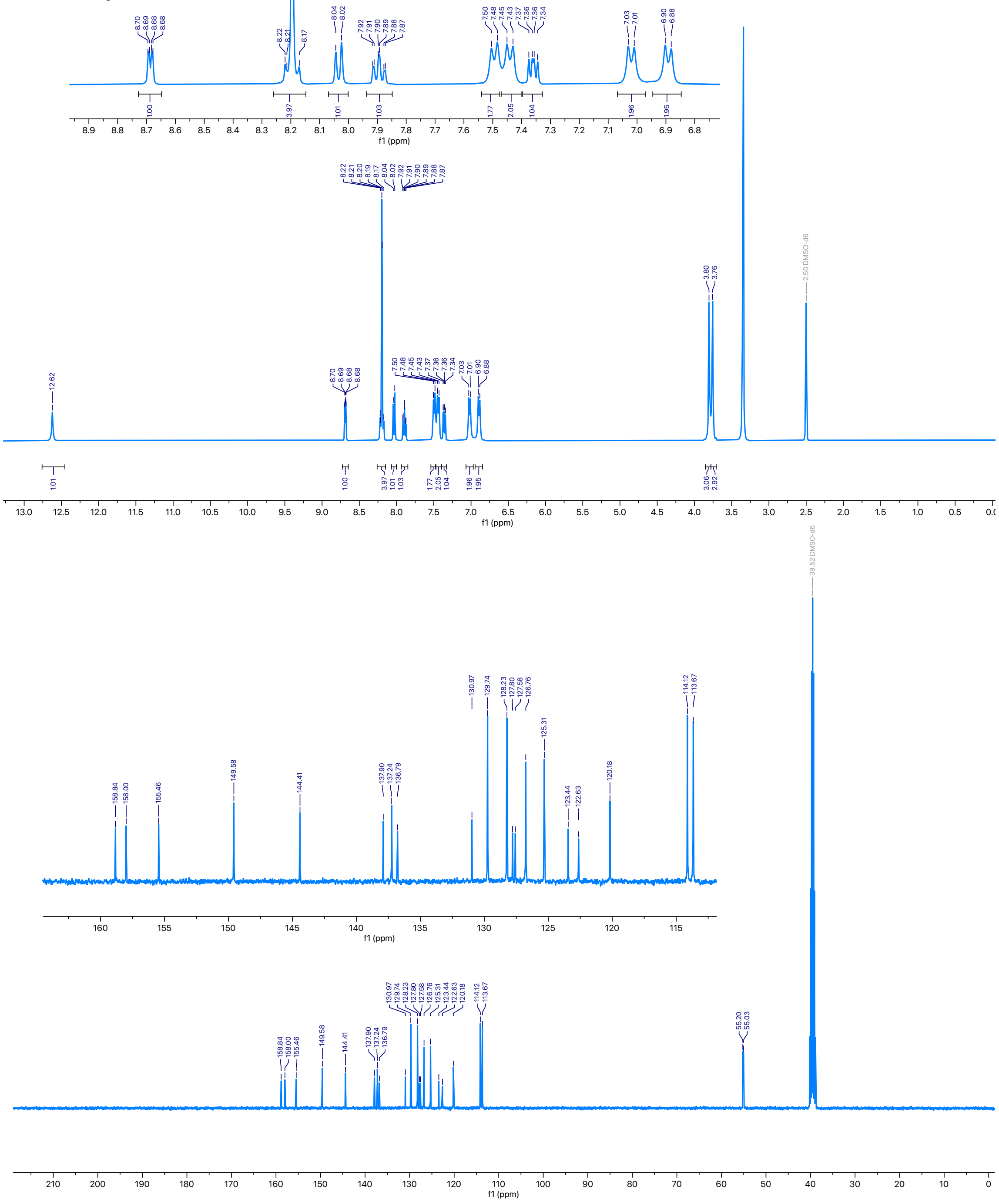

^{1}H (top) and ^{13}C NMR (bottom) spectra of compound **5** in DMSO-d_6 (400 MHz)

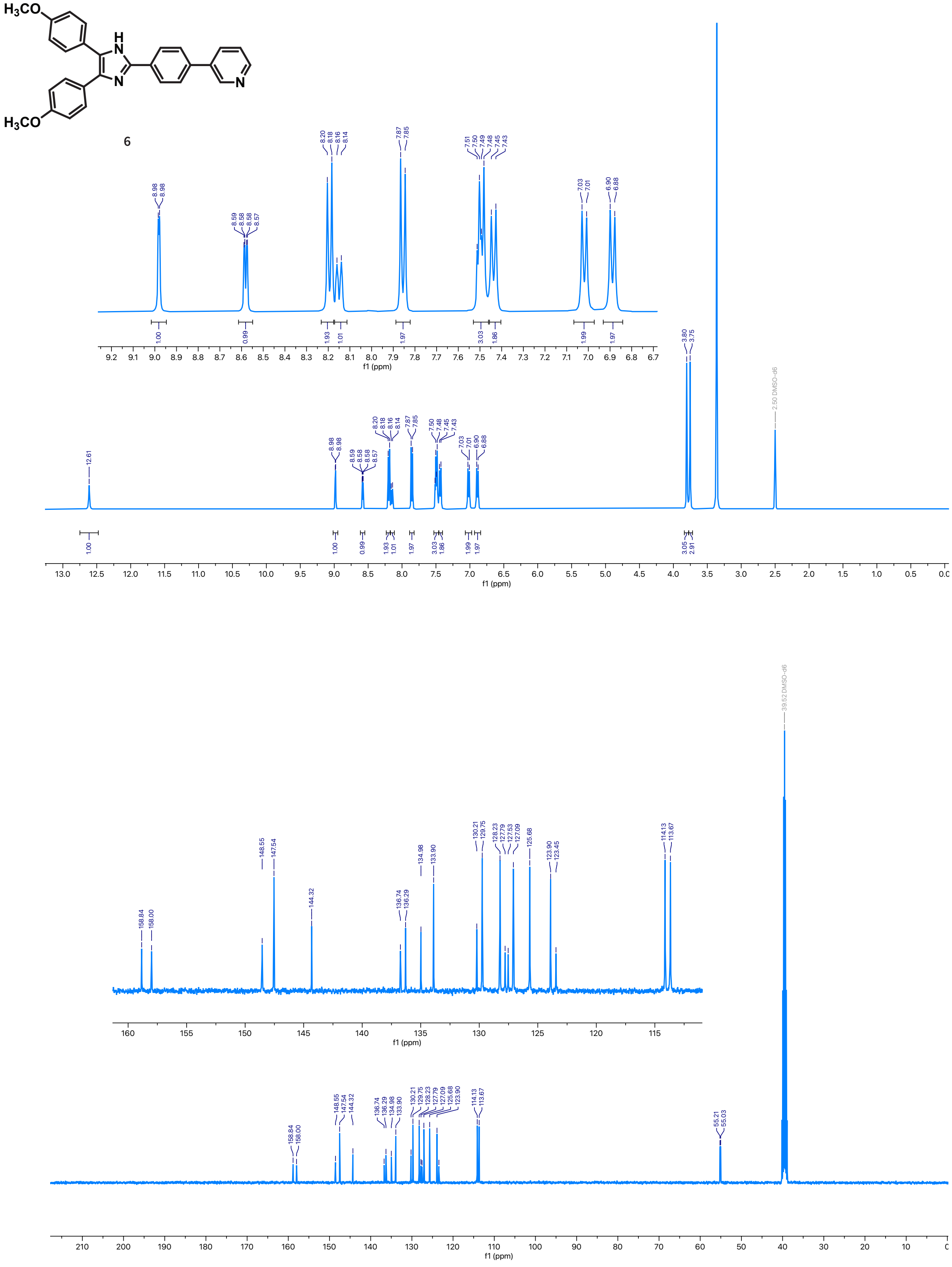

^{1}H (top) and ^{13}C NMR (bottom) spectra of compound **6** in DMSO-d_6 (400 MHz)

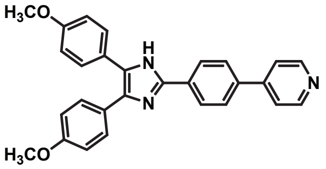

7

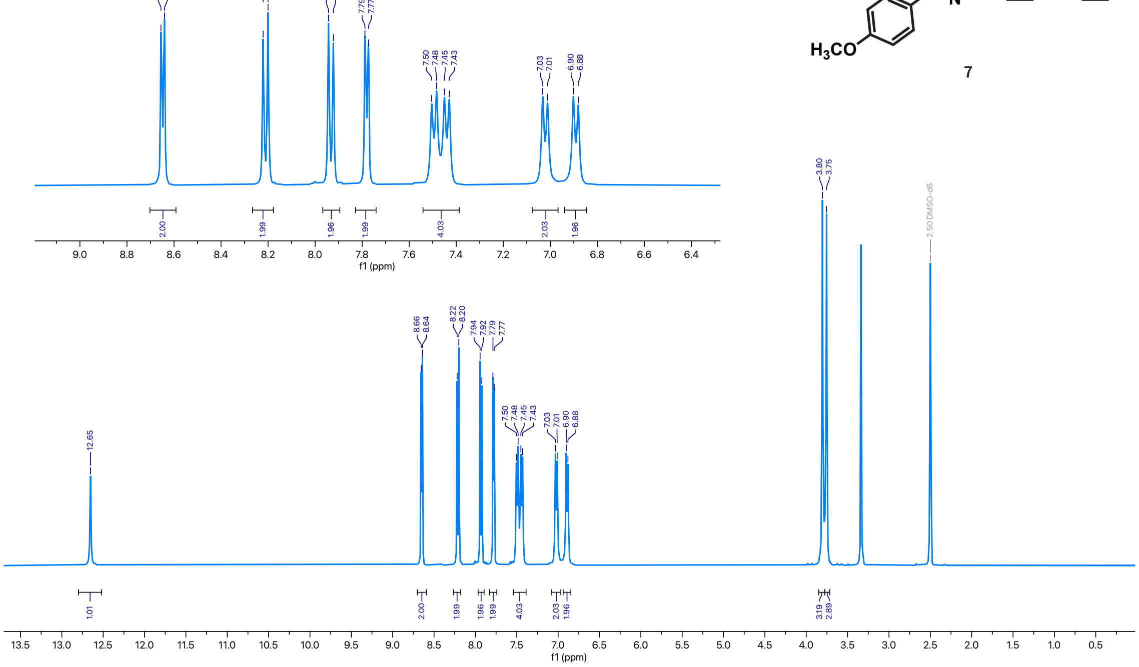

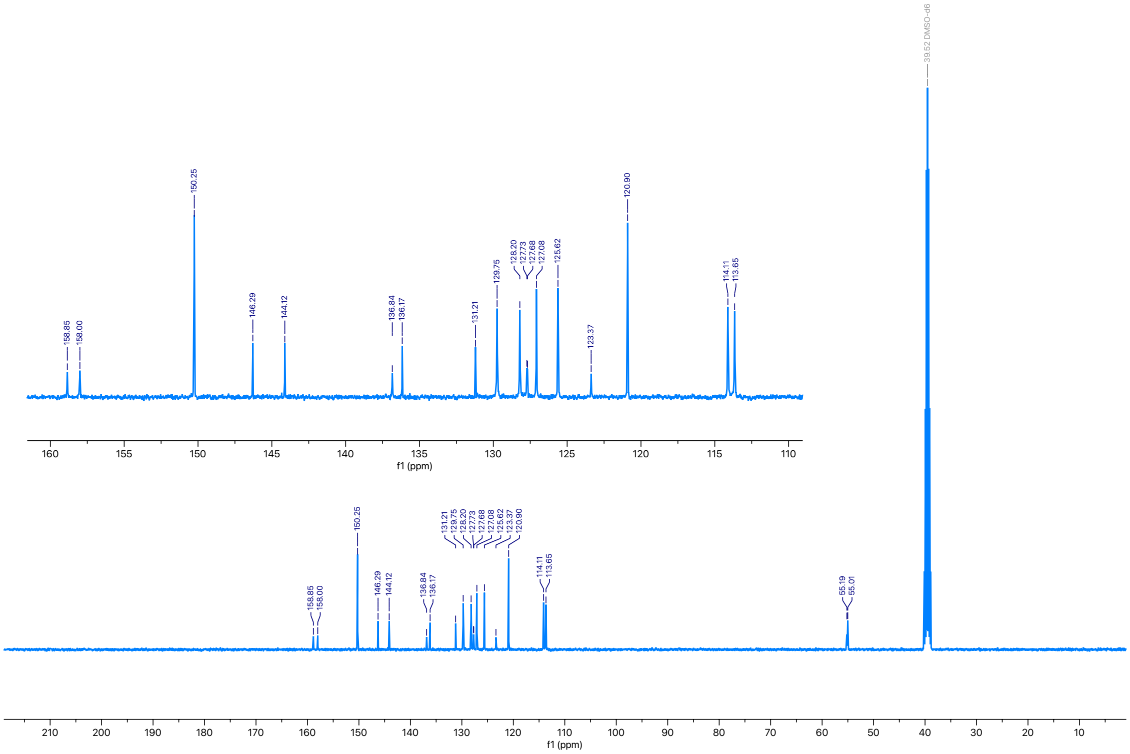

^{1}H (top) and ^{13}C NMR (bottom) spectra of compound **7** in DMSO-d_6 (400 MHz)

4. UV-Vis and fluorescence emission spectra of compounds 1-8

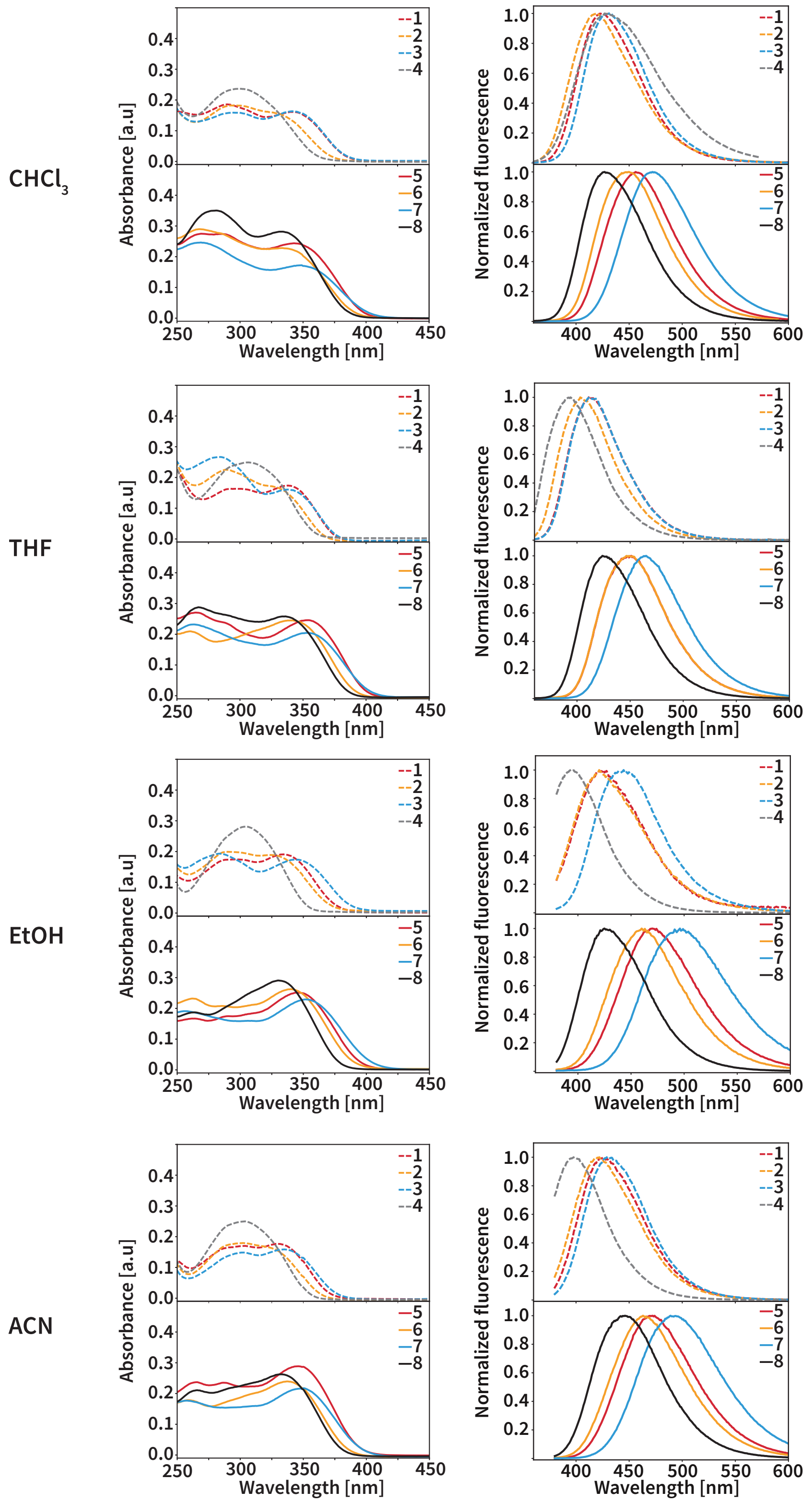

Figure S2. Absorption and fluorescence emission spectra for compounds **1-8** (10 µM) in $CHCl_3$, tetrahydrofurane (THF), ethanol (EtOH), and acetonitrile (ACN)

Table S2. Absorption and fluorescence emission bands of compounds **1-8** (10 µM) in different solvents.

	THF		$CHCl_3$		EtOH		ACN	
Compound	$\lambda_{abs}{}^{a}$ (nm)	$\lambda_{ems}{}^{b}$ (nm)	$\lambda_{abs}{}^{a}$ (nm)	$\lambda_{ems}{}^{b}$ (nm)	$\lambda_{abs}{}^{a}$ (nm)	$\lambda_{ems}{}^{b}$ (nm)	$\lambda_{abs}{}^{a}$ (nm)	$\lambda_{ems}{}^{b}$(nm)
**1**	293, 338	414	**290**, 342	423	296, **334**	422	236, 304, **330**	425
**2**	**289**, 320 (sh)	406	**295**, 320 (sh)	418	235, **290**, 323	421	238, **302**, 318 (sh)	421
**3**	238, **285**, 338	415	295, **341**	431	**237**, 284, 344	442	237, 302, **335**	436
**4**	**235**, 307	396	**300**	429	235, **305**	395	236, **304**	398
**5**	**265**, 354	451	**269**, 286, 343	458	232, 262, 291, **345**	471	238, 266, 287, **346**	472
**6**	261, **342**	451	**268**, 333	450	262, **340**	460	244, 259, **337**	465
**7**	234, **263**, 353	465	**269**, 347	472	234, 256, **352**	495	243, 258, **350**	493
**8**	**267**, 336	427	**281**, 333	427	229, 264, **330**	428	238, 266, **333**	446

[a]The maximum absorption wavelength is depicted in bold, shoulders are indicated with (sh).
[b]Fluorescence emission was recorded upon excitation with the longest absorption wavelength reported in the column λ_{abs}.

5. Solubility of compounds 1-8.

The solubility was estimated via UV-Vis measurements as reported by Kim *et al.* (*J. Am. Chem. Soc.* 2013, 135, 47, 17969–17977). We prepared stock solutions of the compounds at millimolar concentration in ethanol. Then, aliquotes of the stock solution were diluted to concentrations between 1 and 12 μM in Britton-Robinson buffer pH = 6.9, keeping ethanol at 1% v/v. The absorption spectra were recorded in a DS5 double beam UV-Vis spectrophotometer (Edinburgh Instruments).

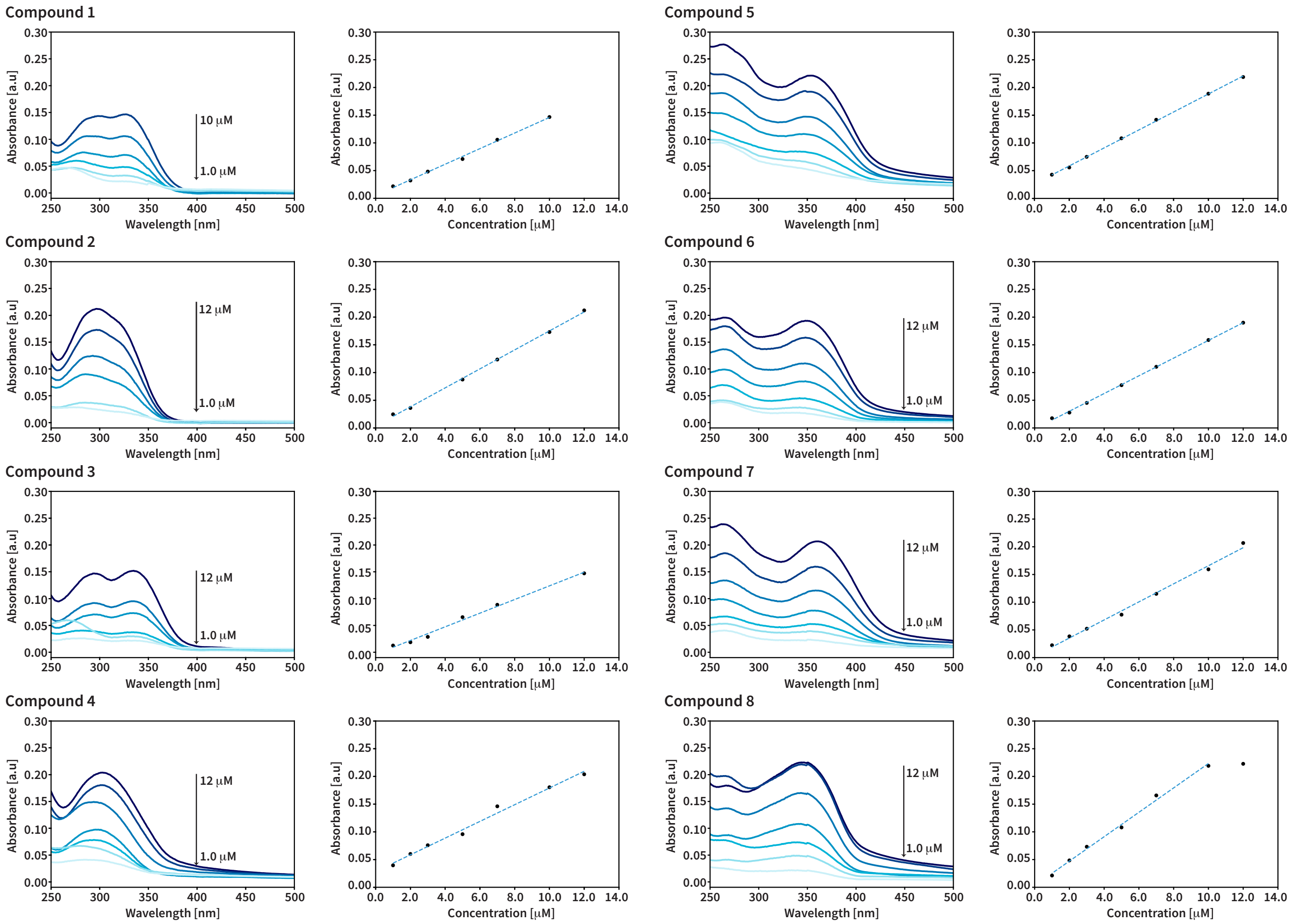

Figure S3. Absorption spectra for compounds **1-8** and plots of absorbance at selected wavelengths against the concentration

6. Photophysical properties of compounds 1-8 under environments of different pH.

Solution of the compounds at concentration between 5 to 10 µM in Britton-Robinson buffer 40 mM with Ethanol 1% v/v at different pH values were prepared. Then, absorption and fluorescence emission spectra were immediately recorded. The longest **λ**$_{abs}$ of each compound was used as excitation wavelength for recording fluorescence emission.

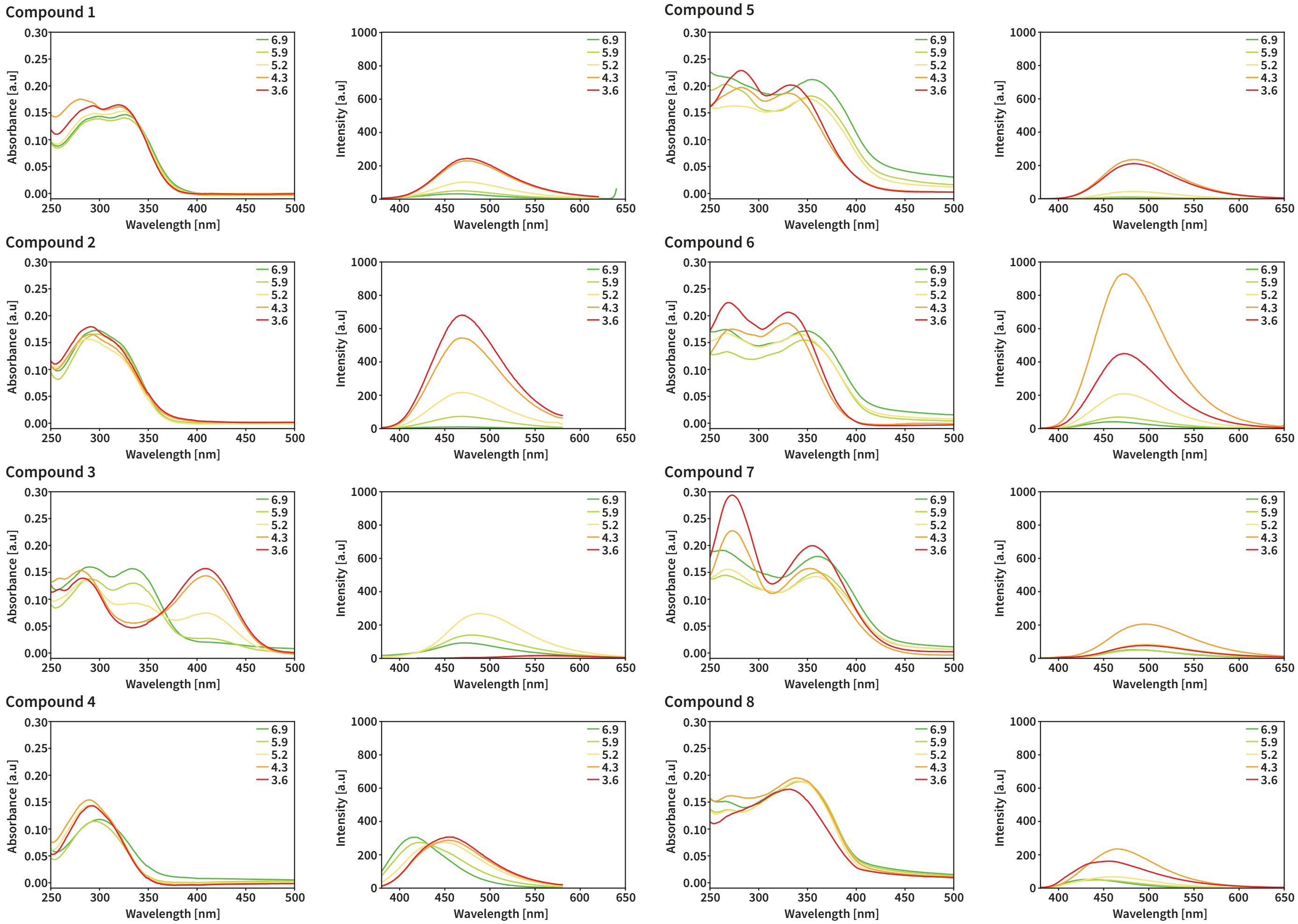

Figure S4. Absorption and fluorescence emission spectra for compounds **1-8** at different pH values.

7. Determination of quantum yield for compounds 1-8.

Fluorescence quantum yields were determined via the relative method. Solutions of the compounds **1-8** were prepared in THF (spectroscopic grade) and in Britton Robinson buffer. Quinine sulfate in 0.1 N H_2SO_4 was used as standard (ϕ_F= 0.54). The absorbance of the solutions was kept lower than 0.1 units at the excitation wavelength. Absorption spectra were measured to determine wavelengths of identical absorption for quinine sulfate and the sample. These wavelengths were used as excitation wavelength. Then, fluorescence emission of these solutions was recorded under identical conditions for sample and standard. The quantum yield was calculated employing the equation 1, where ϕ is the fluorescence quantum yield, *I* is the area under the curve for the fluorescence emission, and *n* is the refractive index of the employed solvents.

$$\boldsymbol{\phi}_{sample} = \boldsymbol{\phi}_{reference}\left(\frac{\boldsymbol{I}_{sample}}{\boldsymbol{I}_{reference}}\right)\left(\frac{\boldsymbol{n}_{sample}}{\boldsymbol{n}_{reference}}\right)^{2} \quad (1)$$

Table S3. Quantum yield of compounds **1-8** in different solvents.

	ϕ_F	
Compound	THF	Aqueous medium[a]
**1**	0.44	0.0030
**2**	0.49	0.0038
**3**	0.39	0.0105
**4**	0.53	0.31
**5**	0.69	0.01
**6**	0.54	0.02
**7**	0.51	0.01
**8**	0.62	0.01

[a]Measured in Britton Robinson buffer pH 7.1 with EtOH 1% v/v.

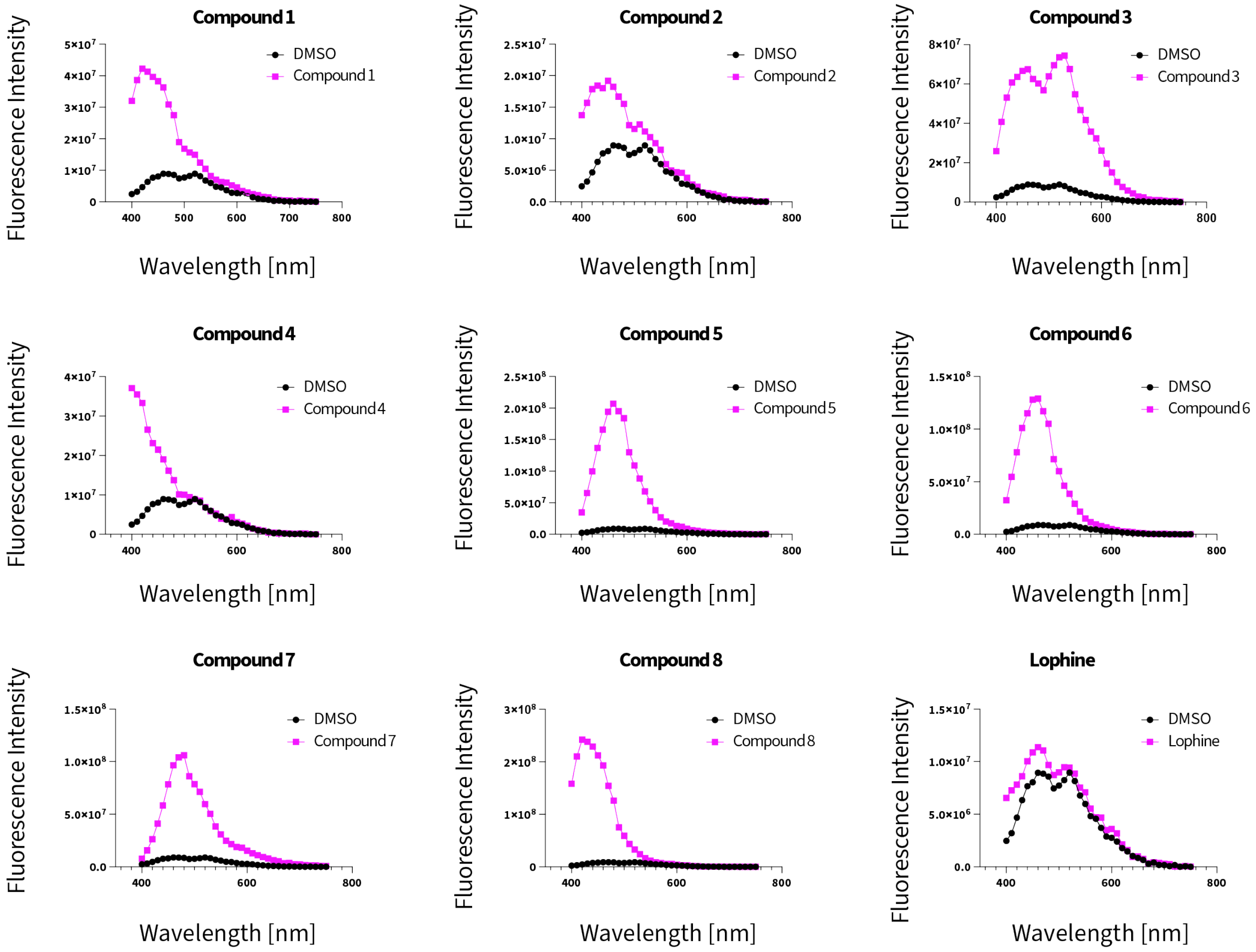

Figure S5. Fluorescence emission spectra for compounds **1**-**8** and lophine were measured with λ_{exc}= 375 nm. Measurements for cells treated with DMSO are shown in black curves. The fluorescence intensity of compounds **1**-**8** in U2OS cells is presented in magenta.

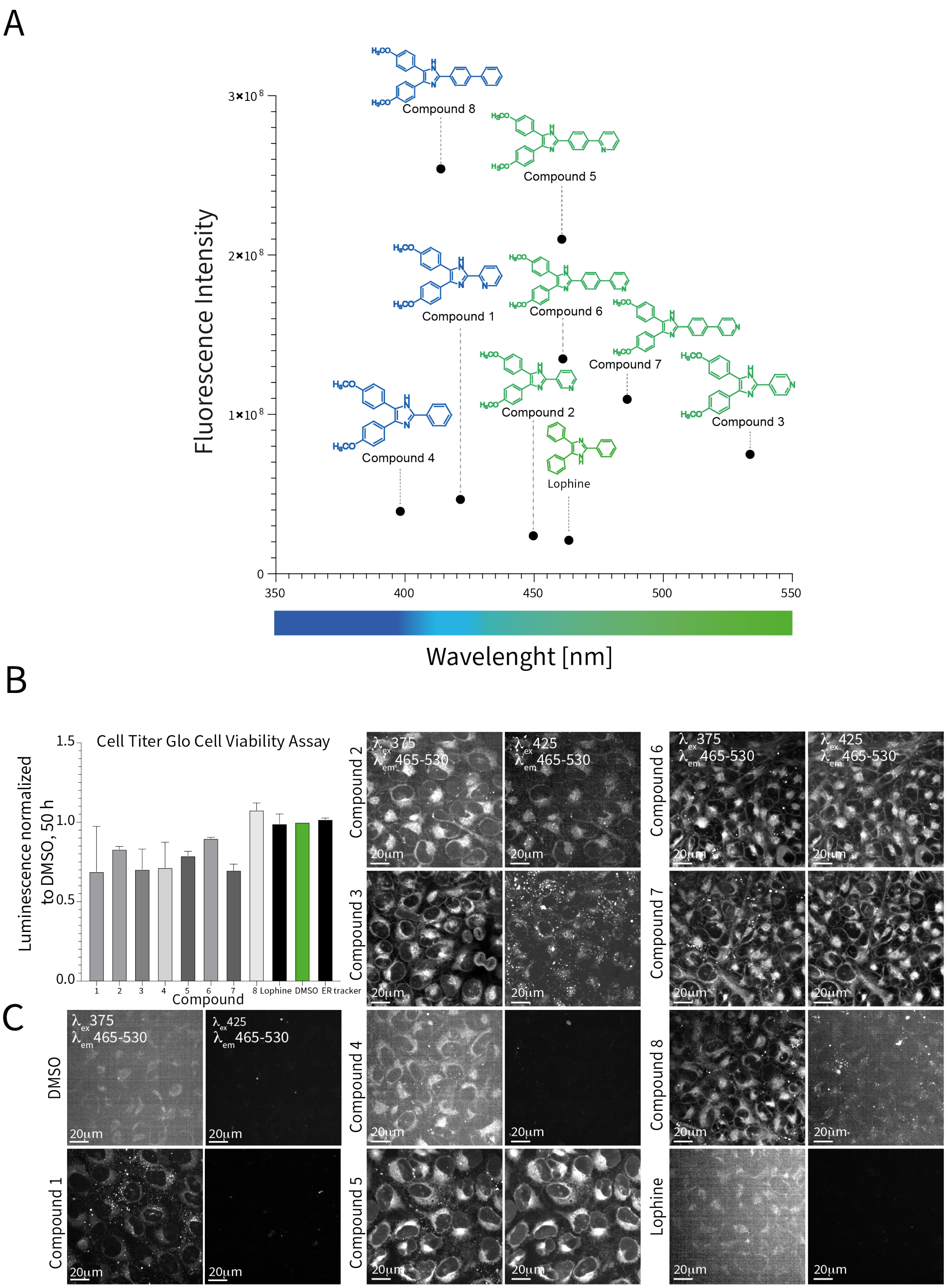

Figure S6. A) Fluorescence intensity and λ_{em} of compounds **1-8** in fluorescence microscopy. B) Normalized luminescence of compounds **1-8** measured by the CellTiter-Glo luminescence cell viability assay in U2OS cells after 50 hours of treatment. C) Confocal images of U2OS cells stained with compounds **1-8** and lophine. Cells were stained with 10 µM of the corresponding compound. Images were acquired using λ_{exc}= 375 nm with λ_{em}= 435-480 nm, and λ_{exc}= 425 nm with λ_{exc}= 465-530 nm. Images were captured using an Opera Confocal microscope with a 40x water objective. Scale bar: 20 µm.

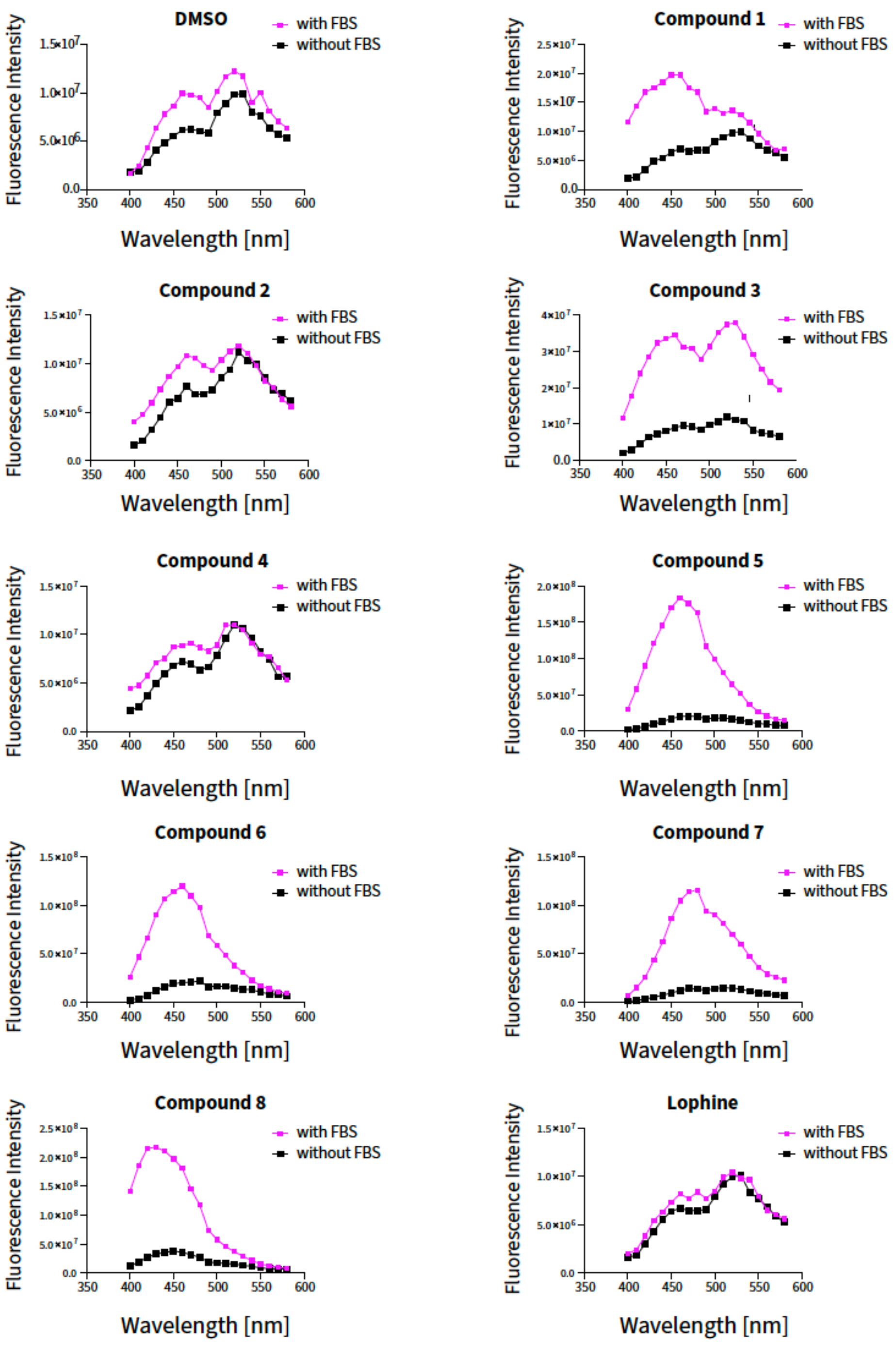

Figure S7. **Biological stability of the compounds in serum.** Cells were seeded and incubated in cell media containing either Fetal bovine serum (FBS) 10% or no serum for 20 hours before staining with 10μM of each compound. Fluorescence intensity spectra were measured using a SpectraMax i3x Multi-Mode Microplate Reader with excitation fixed at 375 nm, recording emission from 405 nm to 580 nm. Our results revealed increased fluorescence intensity in serum-containing media compared to serum-free conditions for compounds **1**, **3**, **5**, **6**, **7**.

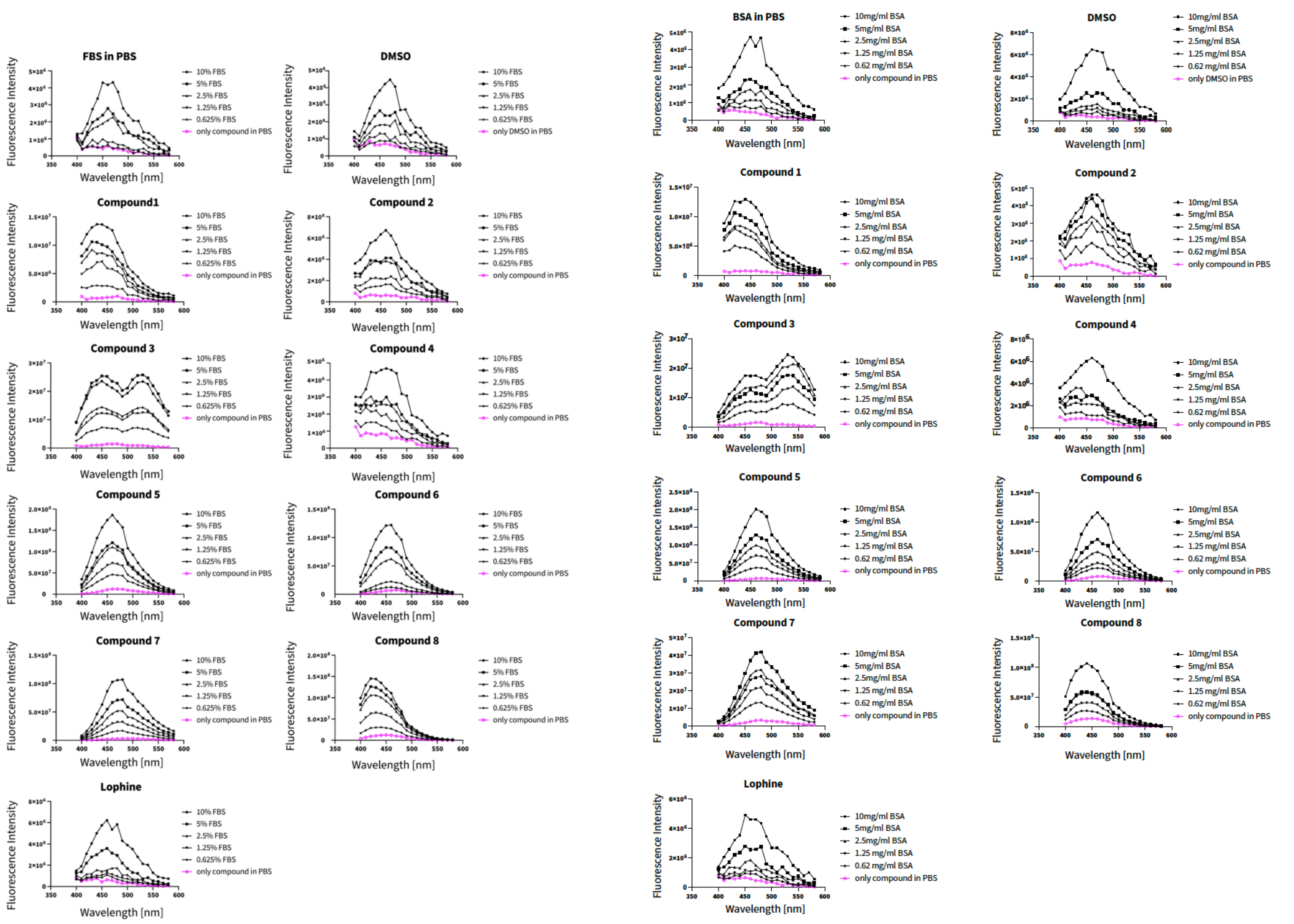

Figure S8. **Biological stability of the compounds in serum.** Fluorescence spectra for the compounds at 10 µM were measured in phosphate buffered saline (PBS) pH= 7.4 containing serial dilutions of FBS (10%, 5%, 2.5%, 1.25%, and 0.625%). In parallel, spectra for the compounds at 10 µM were measured in PBS pH= 7.4 containing serial dilutions (10 mg/mL, 5 mg/mL, 2.5 mg/mL, 1.25 mg/mL, and 0.625 mg/mL) of bovine serum albumin (BSA). Fluorescence intensity spectra were measured using a SpectraMax i3x Multi-Mode Microplate Reader with excitation fixed at 375 nm, recording emission from 405 nm to 580 nm. Increased fluorescence intensity was observed when the concentration of FBS increased. The spectral shape remained unchanged in the presence of serum.

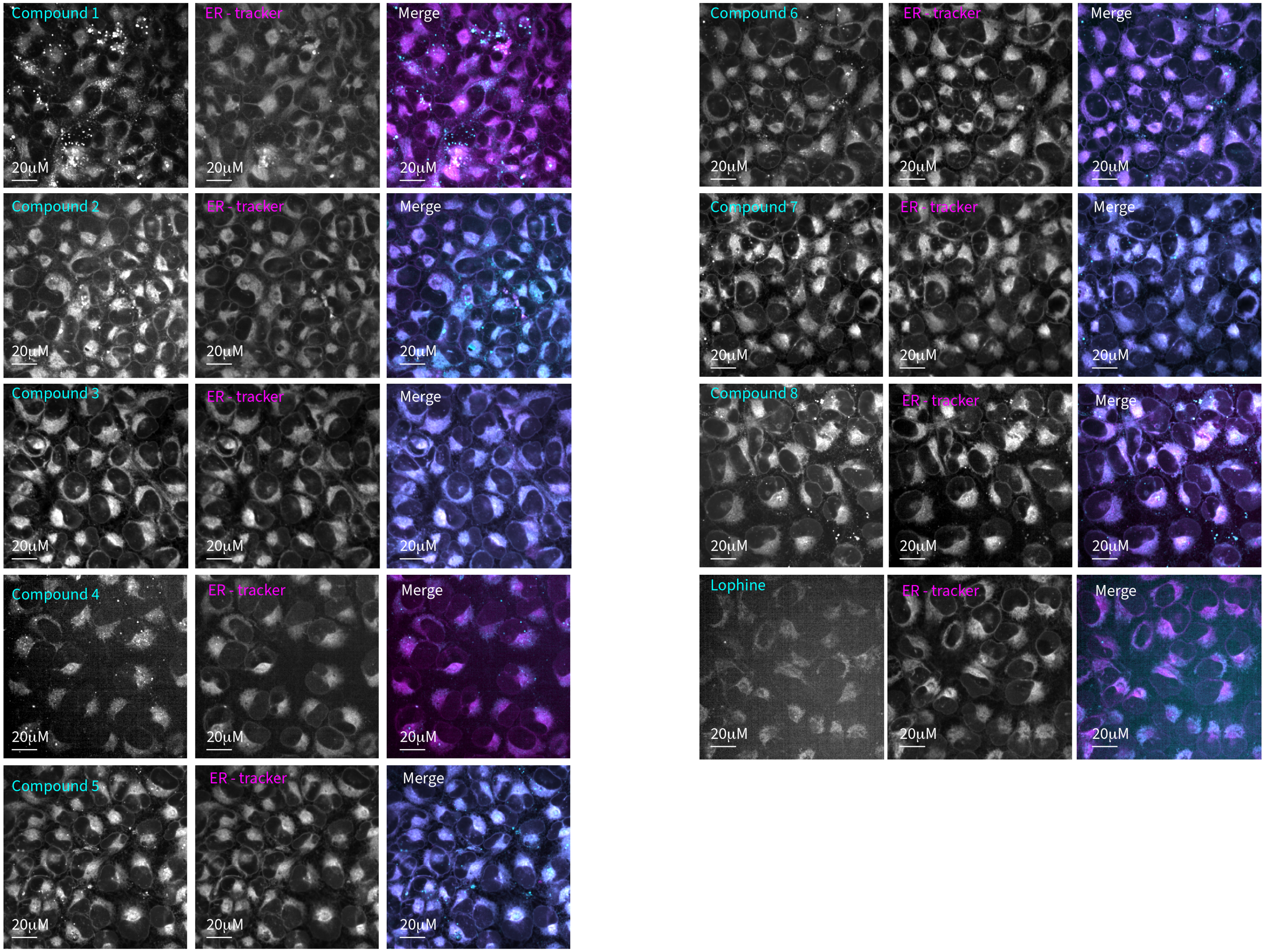

Figure S9. Colocalization staining of U2OS cells treated with compounds **1-8** and ER-tracker. λ_{exc}= 375nm for compounds and λ_{exc}= 561nm for ER-tracker. Scalebar: 20 μm.

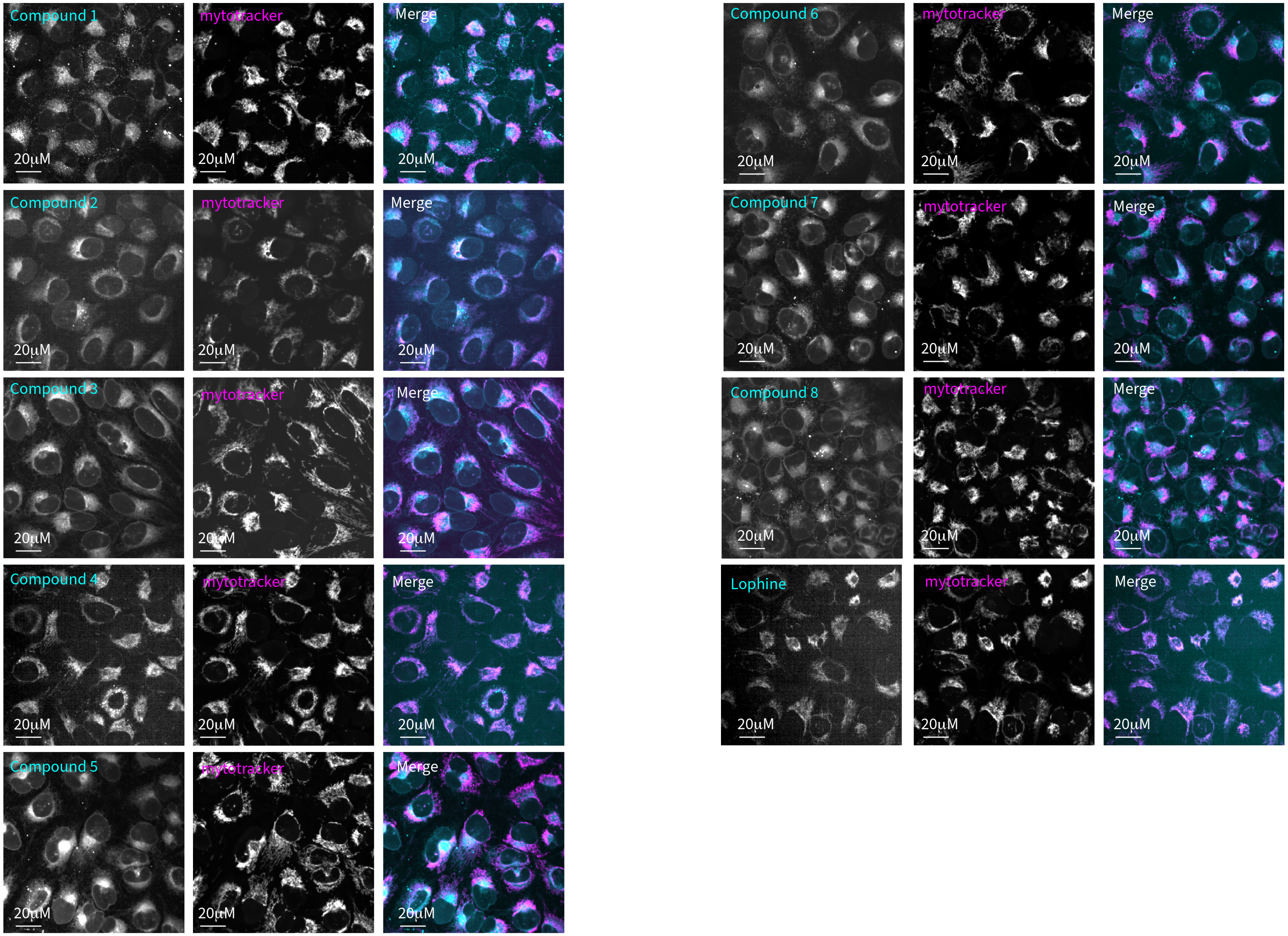

Figure S10. Colocalization staining of U2OS cells treated with compounds **1-8** and Mytotracker. λ_{exc}= 375nm for compounds and λ_{exc}= 640nm for Mytotracker. Scalebar: 20 µm.

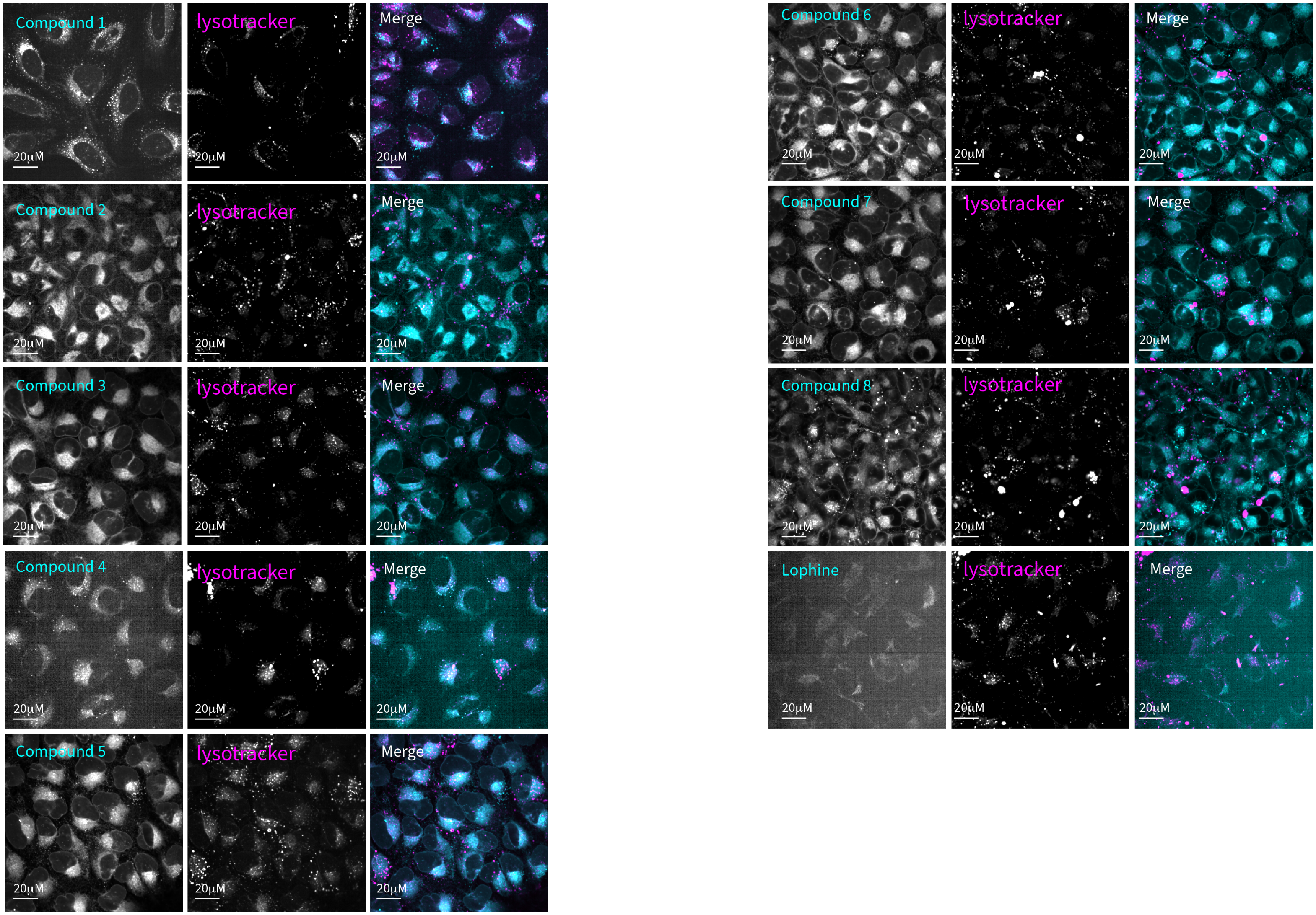

Figure S11. Colocalization staining of U2OS cells treated with compounds **1-8** and Lysotracker. λ_{exc}= 375nm for compounds and λ_{exc}= 561nm for Lysotracker. Scalebar: 20 μm.

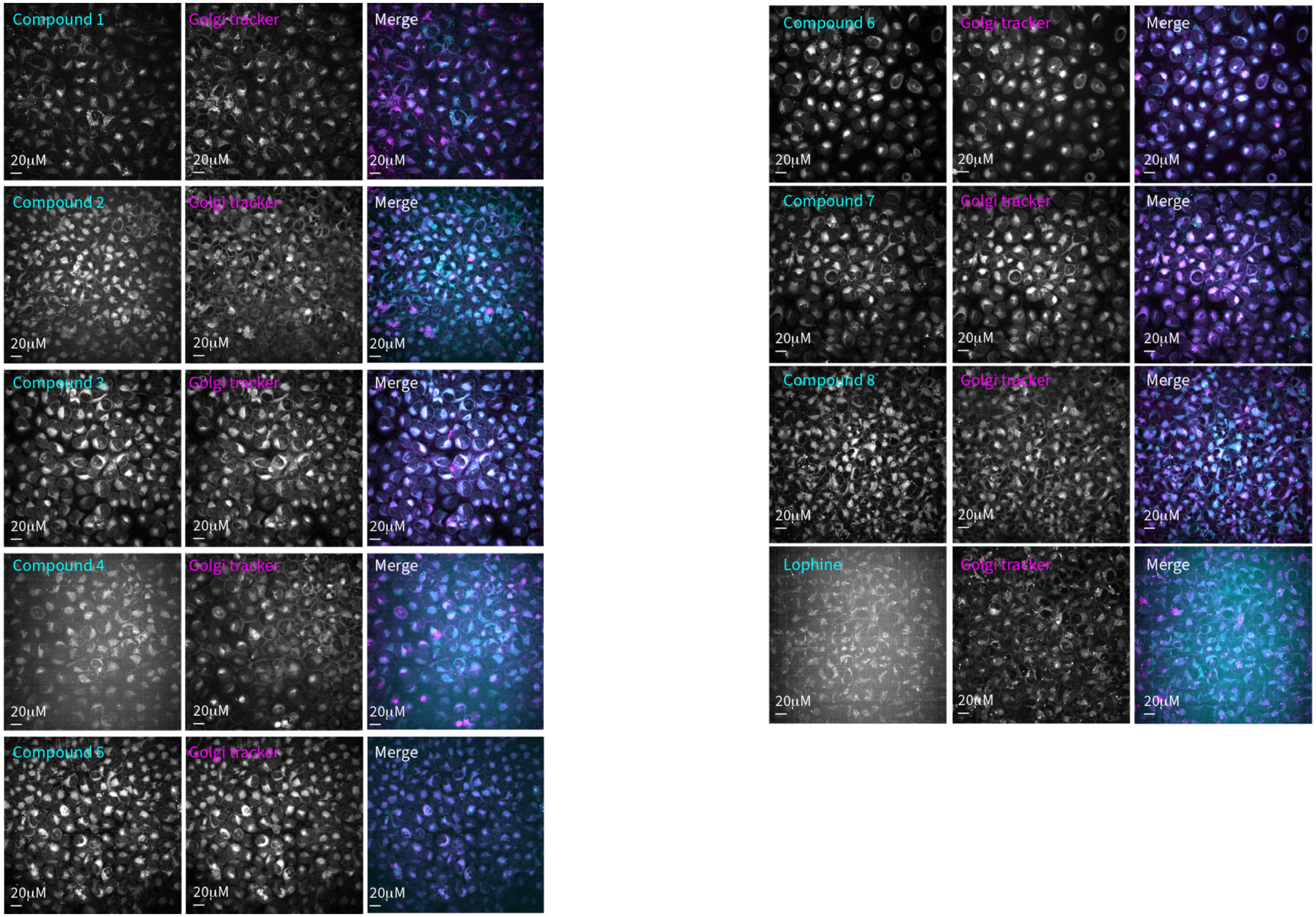

Figure S12. Colocalization staining of U2OS cells treated with compounds **1** to **8** and BODIPY TR- ceramide (Golgi tracker). Excitation wavelength: λ_{ex} 375nm for compounds and λ_{exc}= 561nm for Golgi tracker. Scalebar: 20 μm.

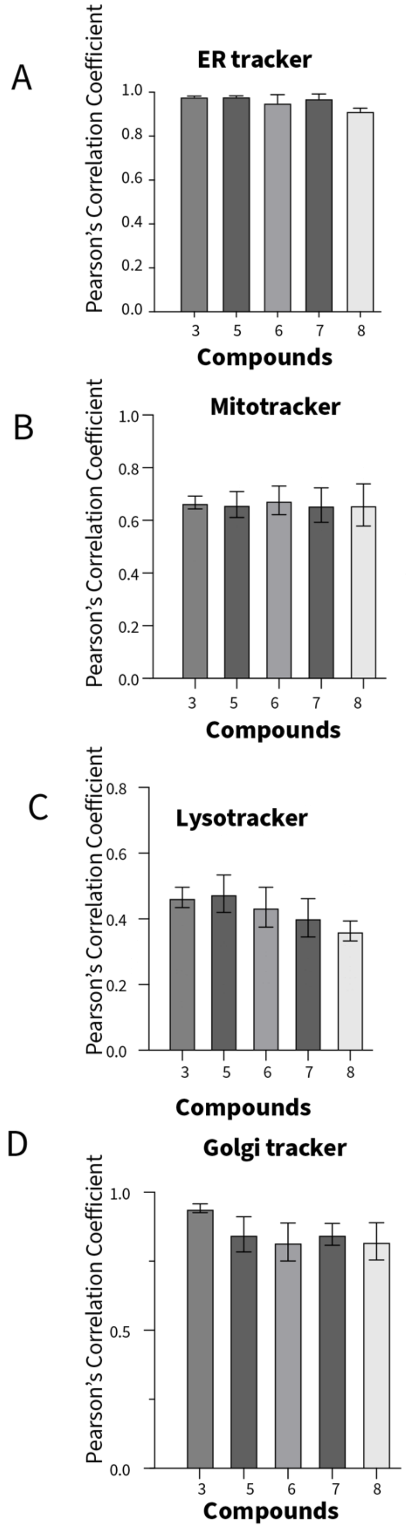

Figure S13. Colocalization measurements for the compounds **3,5-8** with respect to commercial (A) **ER-tracker**, (B) **Mitotracker,** (C) **Lysotracker,** and **(D) Golgi tracker**. Pearson's Colocalization Coefficient was calculated between the two channels (compounds and the commercial tracker) from the confocal images. Measurements were performed using the ImageJ JaCOP plugin, comparing the overlap of signals between two channels.

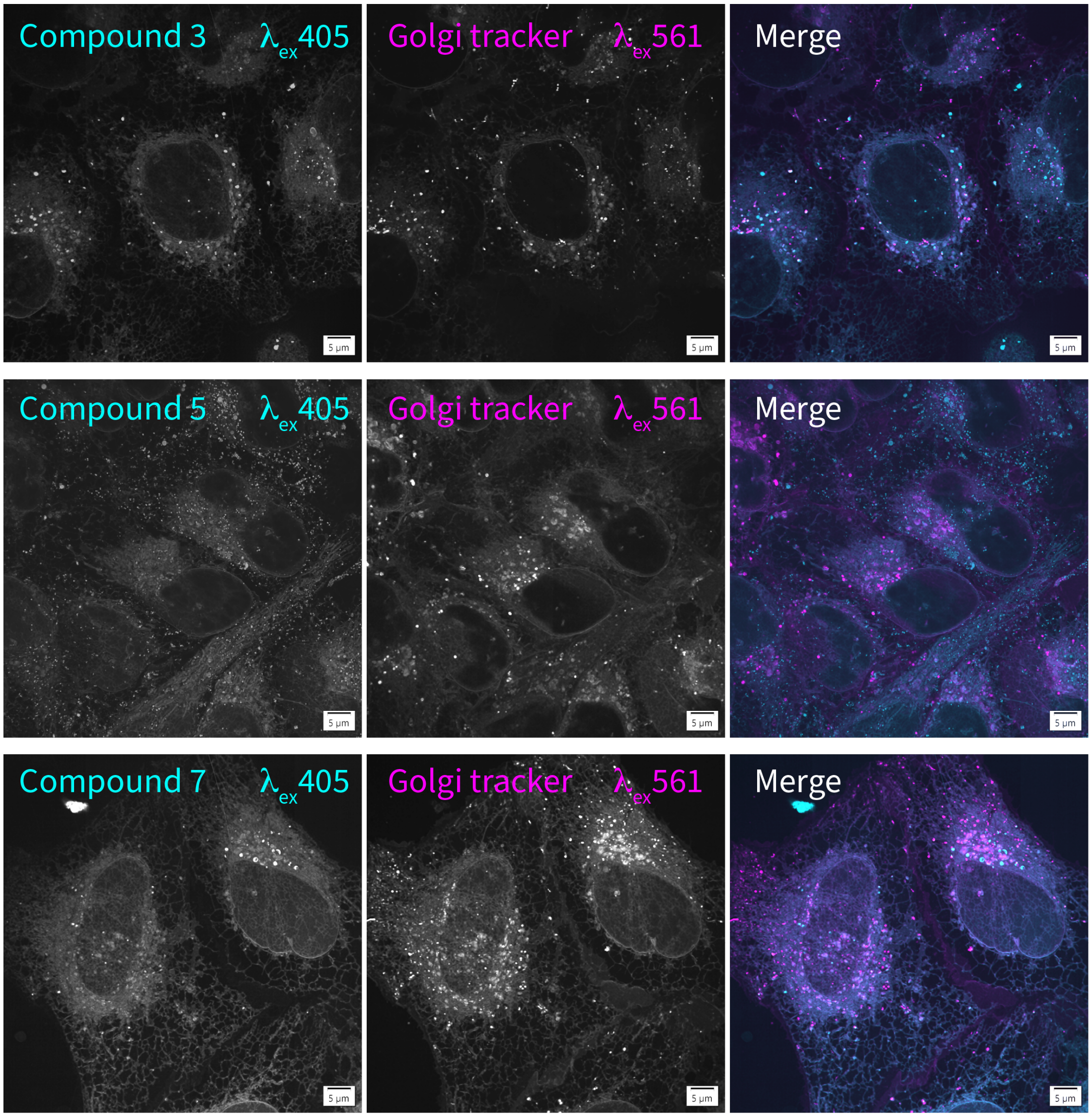

Figure S14. High-resolution confocal images of U2OS cells stained with 10 μM compound **3**, compound **5**, and compound **7** (cyan) and the 2 μM commercial Golgi tracker (magenta). Golgi tracker has shown unspecific staining of the ER and cell membrane. Therefore, we also detect strong colocalization with the Golgi tracker and our compounds. Scale bar: 5 μm.

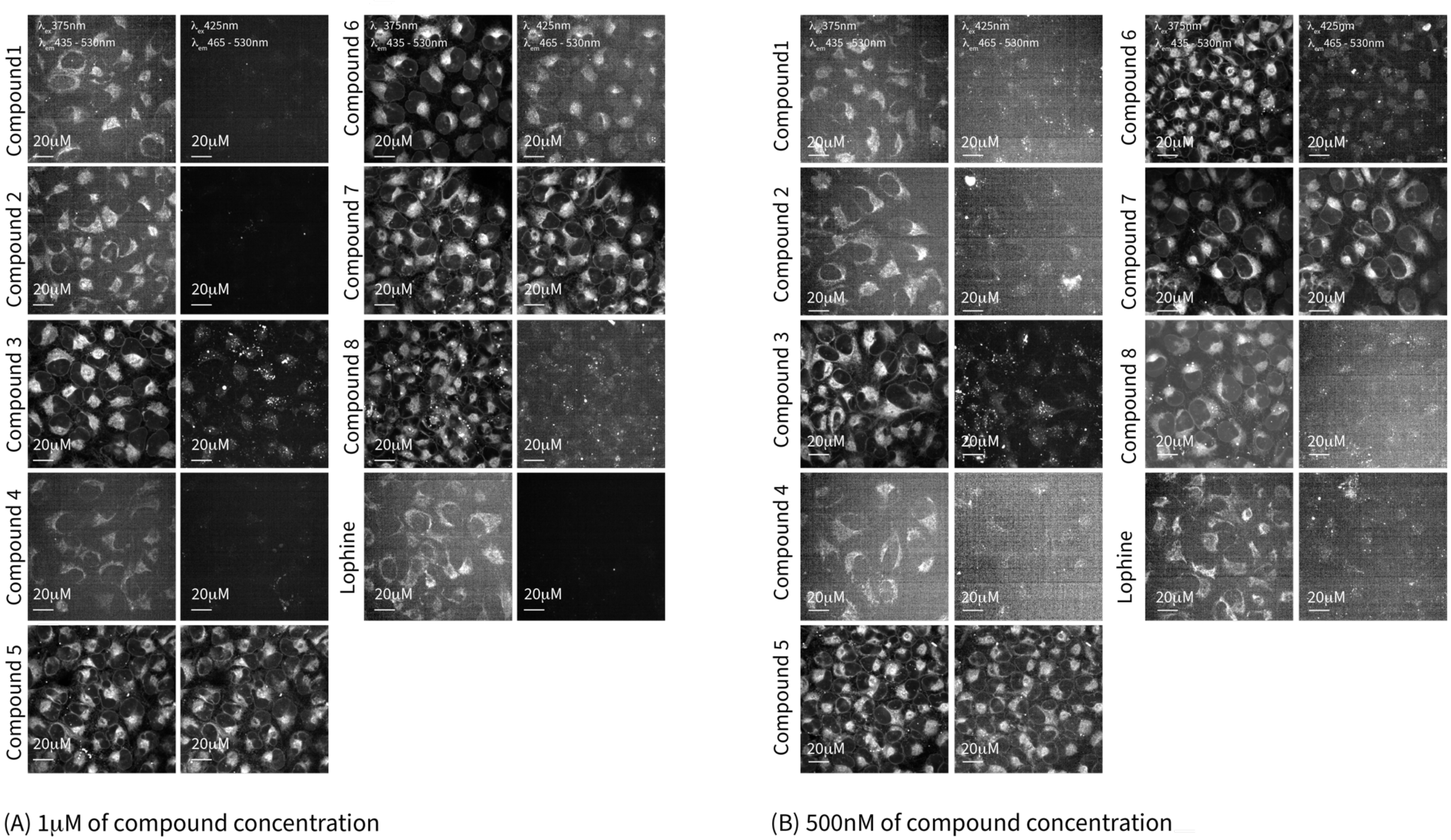

Figure S15. Compounds **1**-**8** exhibit consistent ER staining patterns at both micromolar and nanomolar concentrations. Confocal images of U2OS cells stained with compounds **1**-**8** and Lophine. Cells were stained with 1 μM or 500nM of the corresponding compound. Images were acquired using λ_{exc}= 375 nm with λ_{em}= 435-480 nm, and λ_{exc}= 425 nm with λ_{em}= 465-530 nm. Images were captured using an Opera Confocal microscope with a 40x water objective. Scale bar: 20 μm.

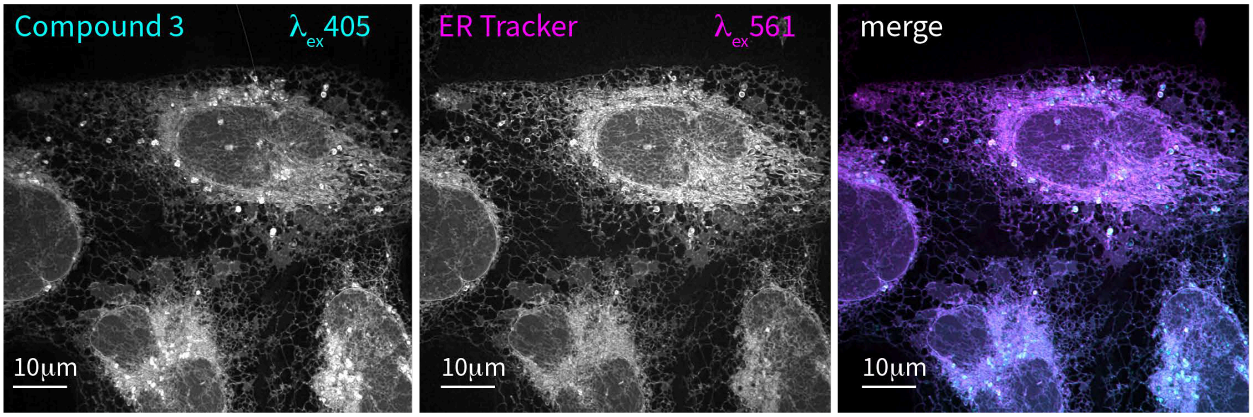

Figure S16. High-resolution confocal images of U2OS cells stained with compound **3** (cyan) and the commercial ER Tracker (magenta) show high colocalization of compound **3** within the ER. Scale bar: 10 µm.

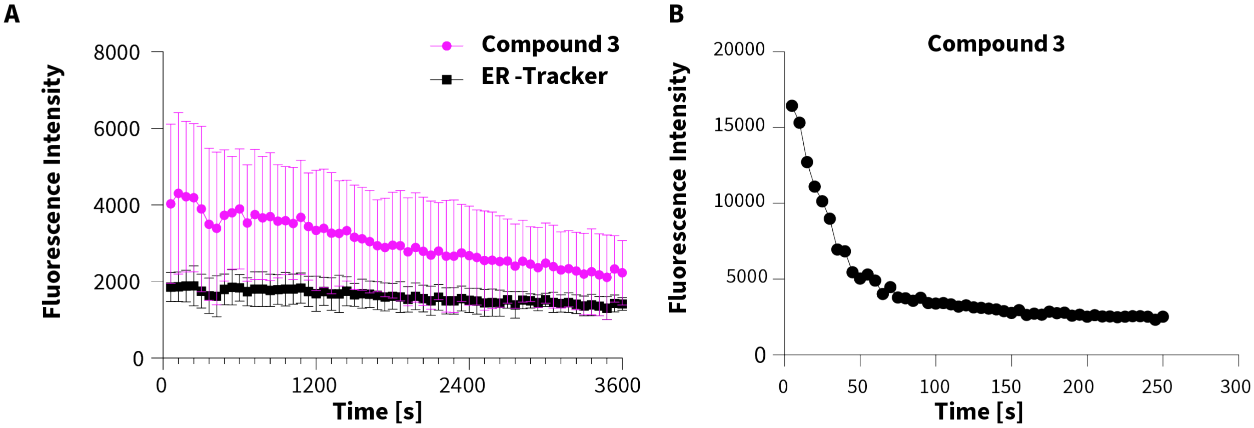

Figure S17. **Photostability of compound 3. A) Photostability of Compound 3 over time compared with ER-tracker.** We recorded fluorescence intensity over time by imaging at one-minute intervals for 1 hour, using BODIPY ER tracker in the red channel as a control. Cells were stained with 10 µM of the corresponding compound and imaged in an Opera Confocal microscope with a 40x water objective with 80ms exposure time and 50% laser power. Images were acquired using λ_{exc}= 375 nm with λ_{em}= 435-480 nm for the compounds, and λ_{exc}= 561nm with λ_{em}= 570- 630 nm for ER-tracker. **B) Photostability of compound 3 over time under more stringent imaging conditions.** Fluorescence Intensity was recorded at 5-second intervals for 300 or 500 seconds. Cells were stained with 10 µM of the corresponding compound. Images were acquired using λ_{exc}= 375 nm with λ_{em}= 435-480 nm (compound **3**) using an Opera Confocal microscope with a 40x water objective with 1s exposure time and 100% laser power.
